# Assessing attention and impulsivity in the variable stimulus duration and variable intertrial interval rodent continuous performance test schedules using noradrenaline receptor antagonists in female C57BL/6JRj mice

**DOI:** 10.1007/s00213-023-06385-9

**Published:** 2023-06-17

**Authors:** L. Klem, M. M. Nielsen, S. B. Gestsdóttir, S. L. Frandsen, S. Prichardt, J. T. Andreasen

**Affiliations:** Dept. of Drug Design and Pharmacology, University of Copenhagen, Universitetsparken 2, 2100 Copenhagen, Denmark

**Keywords:** Attention, Impulsivity, Rodent continuous performance test, Noradrenaline, Antagonist, Arousal

## Abstract

**Rationale:**

Noradrenergic dysfunction is associated with disorders of impulsivity and inattention. The rodent continuous performance test (rCPT) quantifies changes in attention and impulsivity.

**Objective:**

To use NA receptor antagonists to examine the roles of NA on attention and impulsivity behaviours measured in the rCPT variable stimulus duration (vSD) and the variable intertrial interval (vITI) schedules.

**Methods:**

Two cohorts of 36 female C57BL/6JRj mice were examined separately in the rCPT vSD and vITI schedules. Both cohorts received antagonists of the following adrenoceptors: α_1_ (doxazosin, DOX: 1.0, 3.0, 10.0 mg/kg), α_2_ (yohimbine, YOH: 0.1, 0.3, 1.0 mg/kg), and β_1/2_ (propranolol, PRO: 1.0, 3.0, 10.0 mg/kg) in consecutive balanced Latin square designs with flanking reference measurements. The antagonists were subsequently examined for effects on locomotor activity.

**Results:**

DOX showed similar effects in both schedules, improving discriminability and accuracy, and reducing responding and impulsivity, and DOX also reduced locomotor activity. YOH showed prominent effects in the vSD schedule to increase responding and impulsivity, while impairing discriminability and accuracy. YOH did not affect locomotor activity. PRO increased responding and impulsivity, decreased accuracy, but did not affect discriminability or locomotor activity.

**Conclusion:**

Antagonism of α_2_ or β_1/2_ adrenoceptors caused similar increases in responding and impulsivity and worsened attentional performance, while α_1_ adrenoceptor antagonism showed the opposite effects. Our results suggest that endogenous NA exerts bidirectional control of most behaviours in the rCPT. The parallel vSD and vITI studies showed a substantial overlap in effects, but also some differences that indicate differing sensitivity towards noradrenergic manipulations.

**Supplementary Information:**

The online version contains supplementary material available at 10.1007/s00213-023-06385-9.

## Introduction

Disruption of attention and impulse regulation has been associated with dysfunctional dopamine (DA) and noradrenaline (NA) signalling. In attention deficit hyperactivity disorder (ADHD) pathological symptoms of inattention and impulsivity are treated with stimulants or non-stimulants, which broadly enhance catecholamine transmission (Sonuga-Barke et al. 2010; Del Campo et al. 2011; Bluschke et al. 2017; Wolfers et al. 2020). While the evidence of NAergic dysfunction in ADHD patients is less robust relative to DAergic dysfunction, there is evidence that NAergic treatment causes behavioural changes relevant for the psychiatric symptoms (Del Campo et al. 2011). Attentional performance is related to arousal in an inverted U-shaped manner, where both insufficient and excessive DA and NA levels are associated with sub-optimal performances (Arnsten et al. 2007; Arnsten and Robbins 2009). NA shows a higher affinity for α_2_ adrenoceptors and preferentially engages the adrenoceptor at low-to-moderate NA levels, whereas higher NA levels worsen attentional performance through excessive α_1_ and possibly also β_1/2_ adrenoceptor activity (Ramos and Arnsten 2007; Arnsten 2011). A moderate level of α_1_ adrenoceptor signalling is beneficial in certain tasks, including attentional set shifting and sustained attention (Arnsten et al. 1999; Birnbaum et al. 2004; Baldi and Bucherelli 2005; Lapiz and Morilak 2006; Ramos and Arnsten 2007; Berridge and Spencer 2016; Spencer and Berridge 2019). It has been reported that β_1_ and β_2_ receptor subtypes show opposite effects on working memory (Ramos et al. 2005, 2008).

Rodent behavioural assays have been used to examine how catecholamine transmission regulates attention and impulsivity. The recently developed rodent continuous performance test (rCPT) assesses the ability to discriminate between stimuli, by including both target and non-target (S−) stimuli presented in a single fixed location (Kim et al. 2015). This contrasts with the spatially divided attention that is assessed with the five-choice serial reaction time task (5-CSRTT) (Bari et al. 2008) and the five-choice continuous performance test (5C-CPT) (Young et al. 2009). The inclusion of both target and non-target stimuli in the rCPT and 5C-CPT improves translatability to human versions of the CPT. Similar to the human CPT, the rCPT includes measures reflecting discriminability (d′, also called detectability) and response bias (C). The rCPT also measures waiting impulsivity, defined as the tendency to respond before stimulus onset (Voon 2014), in the form of premature responses. Similar to the 5-CSRTT and 5C-CPT, the rCPT enables the use of variable stimulus duration (vSD) and variable inter-trial interval (vITI) schedules. vSD schedules have generally been employed to increase attentional demand, often measured through decreased accuracy or decreased discriminability (Bari et al. 2008; Higgins and Breysse 2008; Callahan et al. 2019), whereas vITI schedules are often used to tax inhibitory control measured as premature responses (Robbins 2002; Bari et al. 2008; Amitai and Markou 2011; Callahan et al. 2019). While the use of a fixed location for target (S+) and non-target (S−) stimuli presentation may improve translatability to human CPTs, the assay permutations differ in the S+/S− presentation ratio. The rCPT schedules used in this study present stimuli at an even 1:1 ratio, while human permutations typically deliver either mostly S+ (e.g., Conners’ CPT; 9:1 S+:S−) or S− stimuli (e.g., X-CPT; 1:5 S+:S−) to bias subjects towards responses (go) or non-responses (no-go), respectively. By not inducing this response bias, there is a lower cognitive demand on rodents in the rCPT relative to humans in the CPTs, e.g., as there is a less pre-potent no-go response. By having an even 1:1 stimulo ratio, the rCPT more closely resembles the rodent go/no-go tasks than clinical CPTs (Kim et al. 2015). However, some human CPT studies have used and even 1:1 probability (Losier et al. 1996), and it has been shown that high S+ probabilities (e.g., 50%) promote a more liberal response strategy, challenging inhibitory control systems (Lynn and Barrett, 2014). Another important consideration is that rCPTs must use a relatively higher S+ ratio than human CPTs, since task engagement of mice is driven by reward and that mice are not inherently motivated to engage in the task.

Our research group recently examined selective and non-selective DA/NA reuptake-inhibitors in the rCPT vSD schedule (Caballero-Puntiverio et al. 2019, 2020), and subsequently in a modified rCPT vITI schedule (Prichardt et al. 2023). The general effect of the NA reuptake inhibitor atomoxetine was to reduce premature responses and increase discriminability in both the vSD (Caballero-Puntiverio et al. 2019, 2020) and in the vITI schedule (Prichardt et al. 2023). The DA reuptake-inhibitor amphetamine decreased premature responses and improved discriminability in the vSD (Caballero-Puntiverio et al. 2019, 2020) and in the vITI schedule (Prichardt et al. 2023), and these effects depended on the baseline performance of the animals (hereafter called reference performance). The examined drugs enhance DA and NA levels and corroborated the role of DA and NA in the regulation of attention and impulsivity. By using competitive NA/DA receptor antagonists, this study examined the specific roles of different DA and NA receptor (R) classes in regulating behaviours in the rCPT. We included both the vSD and the vITI schedules and discuss the results in the context of the effects observed in other behavioural assays. The NA and DA R antagonist results are presented in two separate articles due to the large amount of the collected data, and we refer to the other study for the DA antagonist results (Klem et al. 2023).

## Materials and methods

Two cohorts of 35 and 36 female C57BL/6JRj mice (Janvier, France) were used in this study, aged 7 weeks upon handling and ~10 months upon study completion. The mice were group-housed (four mice per cage) in a controlled environment with a relative humidity of 40–60% and temperature of 20–22 °C. The mice were kept on a regular 12h/12h dark-light cycle with lights on at 8 AM. The cages were supplied with enrichment in the form of wooden sticks, climbing ropes, and red plastic shelters. During the week prior to training, the mice were handled and habituated to both the experimenter and the liquid reinforcement (Yazoo Kids no added sugar strawberry milk). The mice were moved from ad lib to a restricted feeding setup during handling, which maintained their weight down to 85% of ad lib feeding weight, based on available growth curves from Janvier. The mice had free access to water. We housed female mice without male mice in the room, which may lead to the Lee-Boot effect, i.e., suppression or prolongation of the estrus cycle when females are housed together in isolation from males (Champlin 1971; Van Der Lee and Boot 1955). This practice of exclusively housing female mice results in lower levels of fighting or stress compared to males (Fredericson 1952; Scott and Fredericson 1951), contributing to a more ethical laboratory housing practice. Furthermore, the experiments were conducted in the lights phase, as our in-house rCPT data suggests there is a limited impact of light/dark cycle on performance. This observation is supported by a comprehensive review, which found that mice exhibited similar social scores in light and dark phases and that testing in the light phase adequately estimates those obtained in the dark phase (Yang et al. 2008). All procedures were approved by the Danish Animal Experiment Inspectorate, license no: 2017-15-0201-01195, and were conducted in accordance with EU Directive 2010/63/EU and Directive 86/609/EEC for animal experiments.

### The rodent continuous performance test (rCPT)

Our rCPT protocol was based on the original protocol by Kim et al. (Kim et al. 2015), with few modifications, as previously described (Caballero-Puntiverio et al. 2019, 2020; Prichardt et al. 2023). A brief description of the assay, our experiments, and data analysis will be given in the following sections.

#### rCPT apparatus

The mice were placed in touchscreen operant trapezoid-like chambers (Campden Instruments Ltd., Leicester, UK). The chambers were positioned in sound- and light-attenuated boxes. In the front of each chamber there was a touch-sensitive screen covered with a black acrylic mask with three identical cut-outs. The centre of the cut-outs presented the visual stimuli, and ABET II (Lafayette Instruments, IN, USA) recorded, collected, and generated the raw data for subsequent analysis. The experimenters were not blinded to the dosing regimen.

#### rCPT response types, flow, and parameters

In the rCPT, the S+ and S− are presented as a block-randomisation at an even frequency (50%). A hit is designated as a response to the S+ and elicits both a 1-s tone and a 20 μL liquid reinforcement. A miss occurs when the mouse does not respond to the S+. When an S− is presented, a response is classified as a false alarm (also called a mistake), whereas no response is classified as a correct rejection. A mistake will elicit a correction trial, in which an S− is presented again. A response to a correction trial S− (a correction trial mistake) triggers another correction trial, and this loop continues until the animal correctly withholds its response, and then, the regular trials with the 50/50 chance of S+ and S− resume. Note that mistakes and correct rejections obtained during correction trials are not included in the rCPT parameter calculations. A blank screen is shown in between stimuli during the inter-trial interval (ITI). Touches to the blank screen during the ITI are classified as premature responses and trigger an ITI restart correction loop, where the same type of ITI duration, e.g., the 3-s ITI type, will be presented repeatedly until the animal withholds its response for the entire ITI duration. The rCPT parameters are calculated based on these behaviours and are outlined below.

- Hit rate: $$HR=\frac{Hits+1}{Hits+ Misses+1}$$
- False alarm rate: $FAR=\frac{Mistakes+1}{Mistakes+ Correct\ rejections+1}$
- Discriminability (detectability): *d*^′^ = *z*(*HR*) − *z*(*FAR*)
- Accuracy: $$\% Acc=100\%\ast \frac{Hits+1}{Hits+ Mistakes+1}$$
- Response criterion (responsivity)$:C=\frac{-\left(z(HR)+z(FAR)\right)}{2}$
- Premature response level: %PR $=\frac{Centre\ touches\ast 100\%}{Centre\ touches+ Total\ number\ of\ ITIs}$
- First touches level: $\% FiT=\frac{Centre\ touches\ during\ 12s\ ITI\ast 100\%}{Total\ number\ of\ 12s\ ITI s}$

The addition of one in both the denominator and the numerator for the calculations of HR, FAR, %Acc was necessary to ensure that parameters did not equal zero, which would make calculations of d′, C, or further data transformation, impossible.

%PR was calculated for the vSD schedule, whereas premature responses are separated into first touches (FiT) and following touches (FoT) during the ITI restart loops for the vITI schedule, used to calculate %FiT and the FoT/FiT ratio (Prichardt et al. 2023). The study by Prichardt and colleagues indicated that FiTs and FoTs are distinct behaviours and that %FiT in the rCPT provides a more sensitive measure of waiting impulsivity and may translate more directly to %PR in the 5-CSRTT (Prichardt et al. 2023). The FoT/FiT parameter remains to be characterized, and we have included our data for this parameter in the supplementary material to support this characterisation. Furthermore, we included accuracy, which is used to measure attentional performance in the 5-CSRTT, but has not previously been included in rCPT studies. Including accuracy in rCPT studies increases comparability between rCPT and 5-CSRTT research and provides a more nuanced understanding of the effects on attentional performance when described alongside d′.

#### rCPT training and testing

**Fig. 1:**
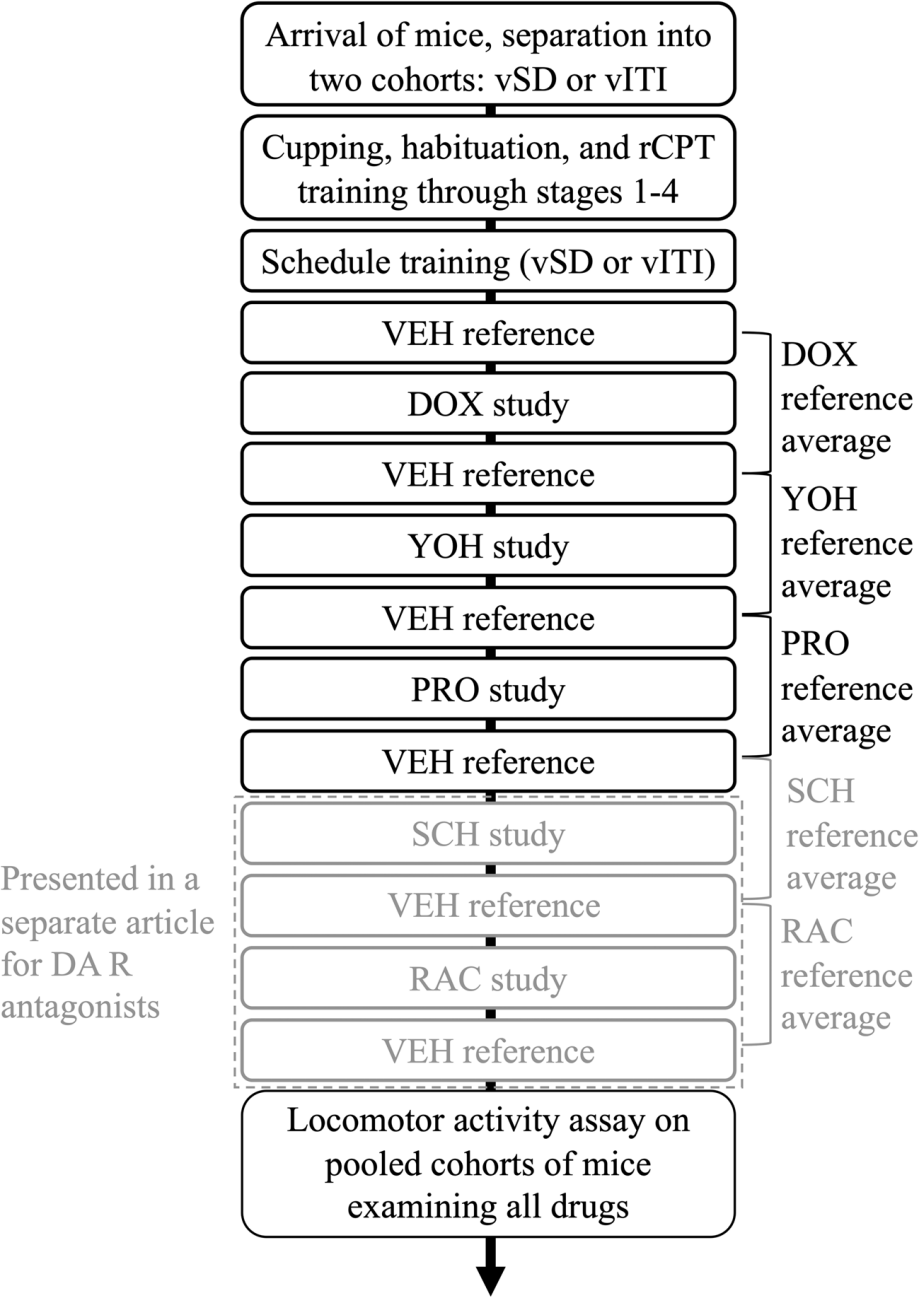
The experimental timeline. When the mice arrived, they were separated into two cohorts, designated for either the vSD or vITI schedule study. The drug experiments were performed in consecutive balanced Latin square designs with reference measurement taken before and after each experiment. For each mouse, the reference level for a given drug experiment was calculated as the average of the two vehicle reference sessions placed before and after the Latin-square design, respectively. We conducted DOX, YOH, PRO, SCH, and RAC drug studies, where the two latter are presented in a separate article, focussing on dopamine antagonists (Klem et al. 2023). The mice were subsequently pooled and examined in a locomotor activity assay examining all drugs and doses, except for 1.00 mg/kg PRO, as described in the relevant discussion section. Abbreviations: rCPT rodent continuous performance test, vSD variable stimulus duration, vITI variable intertrial interval, DOX doxazosin, YOH yohimbine, PRO propranolol, SCH SCH23390, RAC raclopride, VEH vehicle, DA R dopamine receptor

An overview of the experimental process is outlined in Fig. 1.

The mice were initially habituated to both cupping and the liquid reinforcement for 1 week, and food restriction commenced. The mice were habituated to the chamber for a single session, where they consumed 0.3 mL liquid reward from the collection magazine within 20 min. The rCPT training proceeded with five sessions per week and ran for a set duration of 30 min or until the maximum number of rewards was reached, whichever was shorter. The training consisted of four stages gradually increasing in difficulty, which are depicted in Table 1. The two first phases only presented a white square or the target stimulus, S+. An S− was introduced in stage 3, and this was extended to four different non-target stimuli in stage 4 with the introduction of four novel S−. The S+ and S− were presented pseudo-randomly with an equal probability. The intertrial intervals (ITIs) were randomly presented at 2 or 3 s in all stages, and the stimulus duration (SD) was gradually lowered from 10 to 2 s. The limited hold was triggered upon stimulus presentation and set the time in which the mouse could respond to the image, regardless of whether it was still presented. This value was also gradually decreased over the course of the training stages, but generally lasted 0.5 s longer than the SD, until the minimum of 2.5 s was reached. Mice reached baseline training when they had completed stage 4, where they were maintained with a weekly stage 4 (baseline) session, until the remainder of the cohort had completed their training. The vSD schedule cohort received an average of 21 ± 3 (SD) sessions to complete training, while the vITI schedule cohort received 21 ± 5 sessions on average.

**Table 1:**
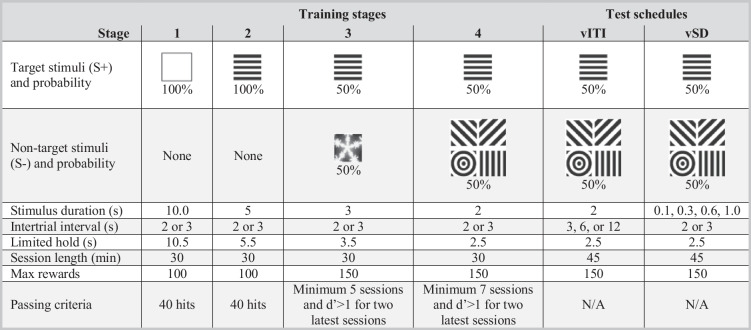
An overview of the four training stages and two test schedules. The target stimuli are exemplified for mice with horizontal lines as the target stimuli, while mice with vertical lines as the stimuli would have these two stimuli interchanged. There was no maximum number of trials

Abbreviations: *rCPT* rodent continuous performance test, *vITI* variable intertrial interval, *vSD* variable stimulus duration. Based on (Kim et al. 2015; Caballero-Puntiverio et al. 2020; Prichardt et al. 2023)

The features of the vSD and vITI testing schedules are also shown in Table 1. The vSD and vITI schedules are considered to place a demand on different behaviours through their schedule design, as described previously. The session length was extended to 45 min for both the vITI and vSD schedules. The vITI schedule shares most of its features with the rCPT stage 4 except for the longer variable ITIs of 3, 6, or 12 s, which increased the likelihood of detecting premature responses while keeping the length of the ITIs unpredictable. A premature response generates ITI restart loops and extends the time between stimuli, delaying the possibility of generating a reward. The vSD schedule also shares the features of the stage 4 setup, except for the shorter and variable stimulus durations (SDs), which were set randomly to 0.1, 0.3, 0.6, or 1.0 s. Despite the brevity of the SDs, the limited hold was fixed at 2.5 s, giving the mice the same amount of time to respond. After training, the animals were habituated to the allocated schedule (vSD or vITI) over a course of five sessions. To minimise the impact of novelty and stress on the experimental outcome, the mice were given a 10 mL/kg subcutaneous saline injection on each of these habituation sessions. Drug testing was subsequently performed twice weekly with a minimum of three days between test sessions. Prior to each test session, stage 4 sessions were performed to confirm that each mouse still met training criteria (d′>1). Testing of each antagonist was conducted in fully counterbalanced individual order according to Latin square designs (LSD). The LSDs contained three doses of antagonist and an interval vehicle measurement, from which the change in response relative to the vehicle condition could be calculated. Each antagonist testing was flanked by reference measurements after vehicle treatment. All compounds were administered subcutaneously in a volume of 10 mL/kg given 30 min prior to testing.

#### rCPT data analysis

The raw data was compiled and analysed using ABET software. HR, FAR, d′, C, %PR, and %FiT were then calculated as described. All statistical analyses were performed in MATLAB (Natick, MA, USA, version 2020b) in a repeated measurements mixed effect model, using the fitlme function for mixed effect models, as described in previous work (Caballero-Puntiverio et al. 2020). The analysis contained the following model:

$$Parameter\sim 1+ dose+ time+ reference+ dose: reference+\left(1\ |\ animal\ ID\right)$$

**Fig. 2:**
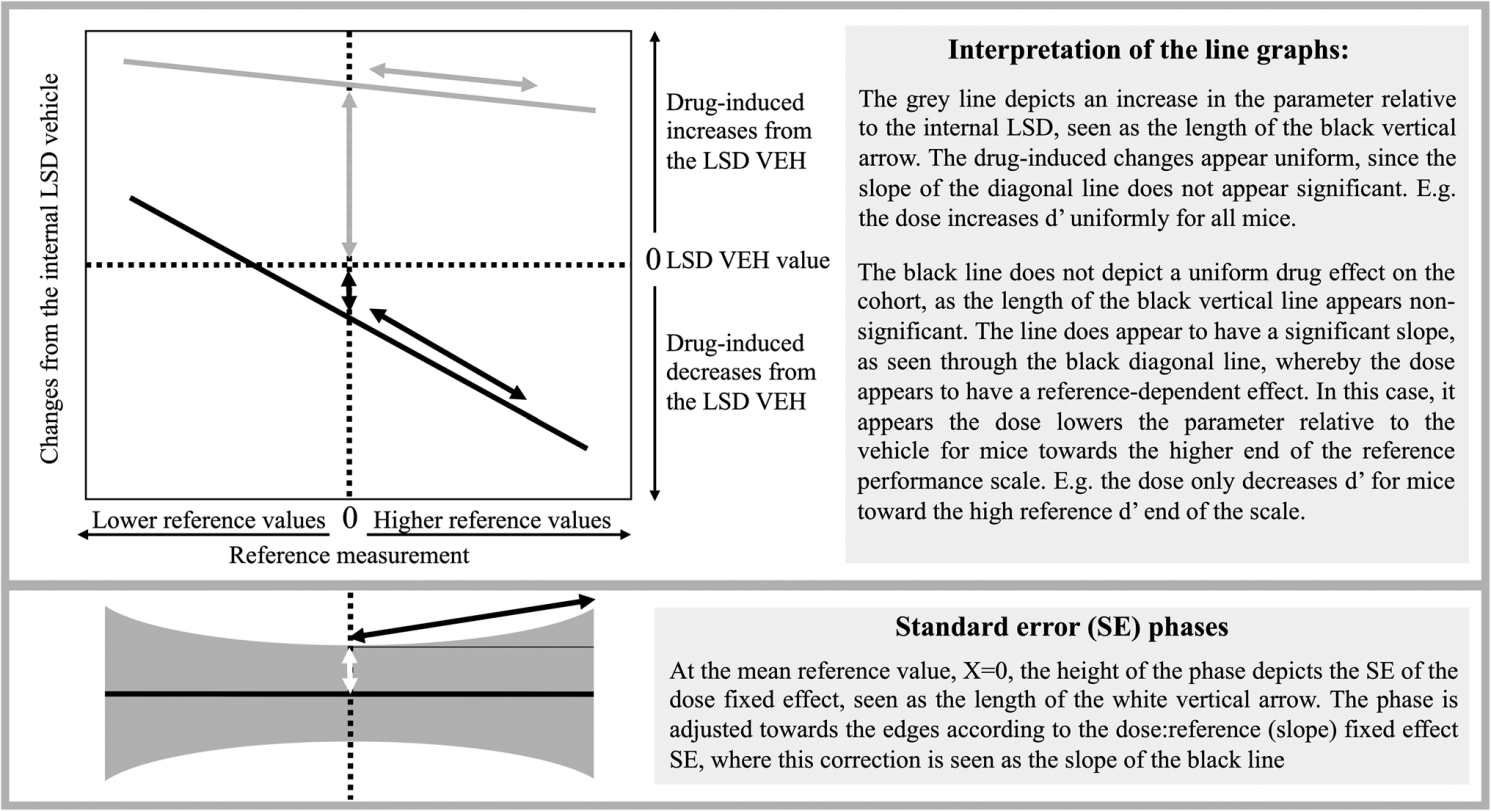
An overview of how to interpret the line graphs presented in the article. Abbreviations: LSD Latin square design, VEH vehicle

Each parameter depends on several fixed effects as well as on the random effect animal ID. The initial “1” in the formula denotes the intercept. Animal-to-animal variation was modelled with a random effect for each animal. The main effects of treatment (dose), time, and reference performance were examined. The model included the principal interaction between each drug dose and reference performance (dose:reference interaction) to ascertain if drug effects were reference-dependent, e.g., if a drug only reduced %PR for mice with high %PR reference values. This interaction was excluded if neither a trend nor a significant effect was detected. The significance calculations for the different terms in the models were based on *F* tests. The individual fixed effect estimates were examined using *t* tests. The data is depicted as both bar charts and line graphs. The bar charts illustrate the observed data without any transformations and were generated using GraphPad Prism (version 9, La Jolla, CA, USA). The bar charts include standard errors of the means (SEM) as error bars. The line graphs were illustrated using MATLAB and depict the modelled data using the most appropriate data transformation to comply with the analysis assumptions, which will be elaborated in the next paragraph. The line graph Y-axis indicates response to the drug relative to the internal LSD vehicle measurement (the dotted line at y=0). The x-axes in the line plots arrange the mice relative to the average reference performance for the entire cohort, i.e., the mean value of the cohort is set to X=0. These two elements allow for detection of both the overall dose effects on the cohort and for reference-dependent effects (dose:reference interaction). The average dose effects are estimated as the distance on the Y-axis between the line and the X=0 point, as illustrated in Fig. 2. The line graphs include standard error phases surrounding the lines. The width of these phases at X=0 corresponds to the model-estimated standard error of the mean for the dose fixed effects, which are then adjusted towards the edges of the line based on the standard error of the slope, as depicted in Fig. 2. Results were considered significant for *P* values below 0.05, with significance presented as follows: *P*<0.05*, *P*<0.01**, *P*<0.001***. Trend values, *P*<0.1, were indicated with asterisks in parenthesis, (*). Note that asterisks were used to describe significant overall dose effects, while slope effects were denoted with a hashtag, #. In the hypothetical Fig. 2, the grey line may have a significant overall dose effect, e.g., *, but not a significant dose:reference interaction (slope), whereas the black line may not have a significant overall dose effect, but a significant dose:reference interaction, e.g., #.

Where appropriate, the rCPT parameters were transformed to comply with the assumptions of the analysis. Logit transformation was applied to prevent confounding by floor- or ceiling effects when analysing percentage and rate data (HR, FAR, %Acc, %PR, and %FiT). The logit transformation is calculated as: $\mathit{\ln}\left(\frac{Y- Lower\ limit}{Upper\ limit-Y}\right)$, where Y is the observed value, and the lower or upper limits refer to the theoretical values. Logit transformation adds weight to values approaching the theoretical limits. With this transformation, a 5%-point reduction in %PR, for example, in two mice with a vehicle value of e.g., 6% and 35%, respectively, would count as a larger effect in the former (a reduction from 6% → 1% is proportionally larger than a reduction from 35% → 30%), rather than appear as a uniform effect in both. The theoretical limits used in the logit transformations are described further in the supplementary material. The calculations of d′ and C were unconstrained by ceiling and floor effects and did not require transformation. The validity of the statistical approach and test-retest stability were analysed in correlations analyses of the reference values, shown in the supplementary material (“Test-retest reliability”).

The cohort size of 36 mice was based on previous research within our group using the line graph analysis approach, which generally requires a minimum of 30 mice to ensure a straight line can be drawn from the mice with low values on one end of the x-axis towards the mice with high reference values on the other end of the scale (Caballero-Puntiverio et al. 2020, Prichardt et al. 2023).

### Locomotor assay setup and analysis

The locomotor activity (LA) assay was included to assess non-specific locomotor-stimulant or -depressant effects of the antagonists, as such effects may confound the interpretation of the rCPT behavioural outputs. LA testing was conducted in a dimly lit room using transparent type III H cages (L × W × H: 42.5 × 26.5 × 18 cm) on a light background. The cages were covered with regular plastic wrap with a few airholes to discourage jumping and escaping. LA was recorded by a camera mounted in the ceiling and connected to a computer with EthoVision XT (Noldus) software. The mice were not acclimatised to the room prior to testing, but were allowed 5 min of habituation to the testing arena before the activity was recorded. All compounds were administered subcutaneously in a volume of 10 mL/kg 30 min prior to testing, which began 5 min after placement of a mouse in the test arenas and was recorded for 40 min. The arenas were cleaned between mice. Each mouse was tested twice with a minimum of 7 days between testing. The treatments were balanced so that each mouse received different drugs, and all doses were represented on each test day. The raw data was recorded and collected by the EthoVision XT (Noldus) software and analysed using GraphPad Prism (version 9, La Jolla, CA, USA). Treatment effect of the total distance travelled was analysed with a one-way analysis of variance (ANOVA), followed by post hoc planned pairwise comparisons with the vehicle control treatment, using Dunnett’s test. Results were considered significant for *P* values below 0.05. Trend values, 0.05<*P*<0.1, were also reported in parenthesis. Data were log-transformed before analysis to comply with the ANOVA assumptions of variance homogeneity, but the results are presented on the original scale.

### Pharmacological interventions

The drugs were administered subcutaneously, and all solutions were adjusted to pH = 7±1. Doxazosin mesylate (DOX 1.00, 3.00, and 10.00 mg/kg) was purchased from Adooq. Yohimbine hydrochloride (YOH 0.10, 0.30, 1.00 mg/kg) was purchased from Tocris. Propranolol hydrochloride (PRO 1.00, 3.00, 10.00 mg/kg) was purchased from Medchemexpress. DOX was dissolved in a 5% D-glucose and 5% hydroxypropyl-beta-cyclodextrin vehicle, while the vehicle for the other drugs contained 0.4% dimethyl sulfoxide and 0.9% sodium chloride. The dose-ranges used in the studies were based on a literature review and on pilot dose-finding studies described in the supplementary material for this article (“Dose-finding pilot study”).

## Results

**Fig. 3:**
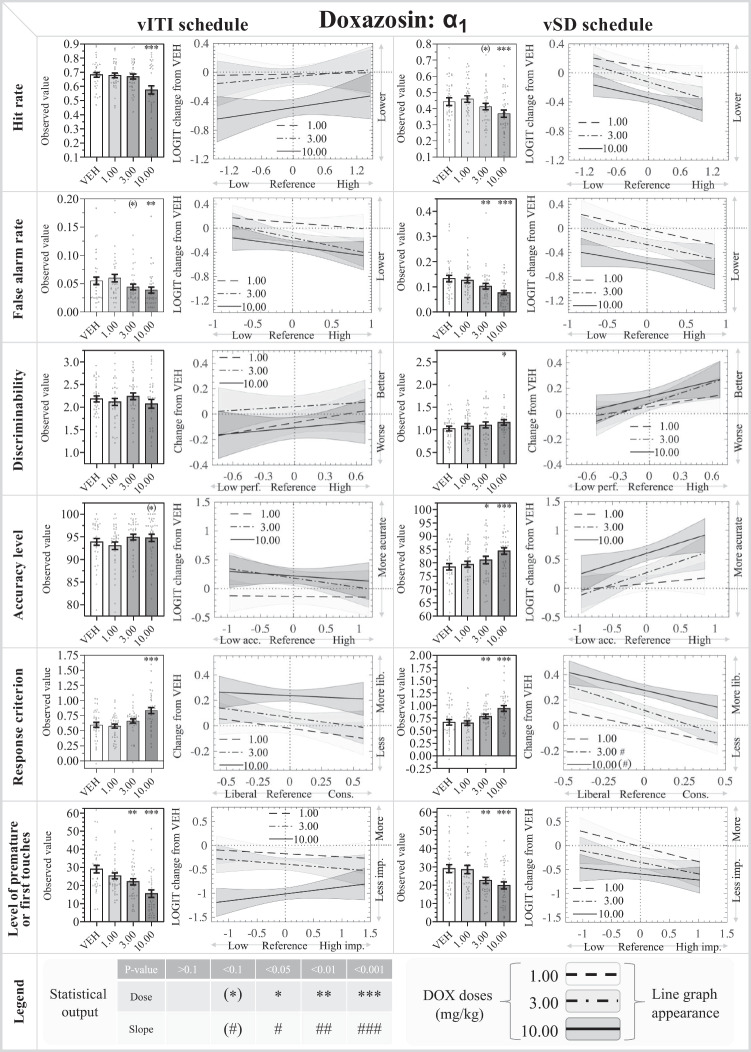
Results from the α_1_ adrenoceptor antagonist: doxazosin (DOX: 1.00, 3.00, 10.00 mg/kg) in the rodent continuous performance test. The left- and right-hand sides show the data from the vITI and vSD schedules, respectively. The bar charts depict the observed data on the original scale, while the line charts depict the modelled reference-dependent effects as analysed data with the appropriate transformations. The line chart y-axes denote changes relative to the within-subject VEH measurement within the Latin square design. The zero value on the x-axis indicates the mean of the reference values for the whole cohort. Significant reference-dependent treatment effects are reflected by line slopes that significantly differ from 0. The line graphs include a shaded standard error phase, depicting the modelled standard error of the dose at X=0, modified by the standard error of the slope towards the edges. Abbreviations: *vSD* variable stimulus duration, *vITI* variable intertrial interval, *VEH* vehicle, *FAR* false alarm rate, *imp.* impulsivity, *cons.* conservative. N for vITI and vSD: 36

**Fig. 4:**
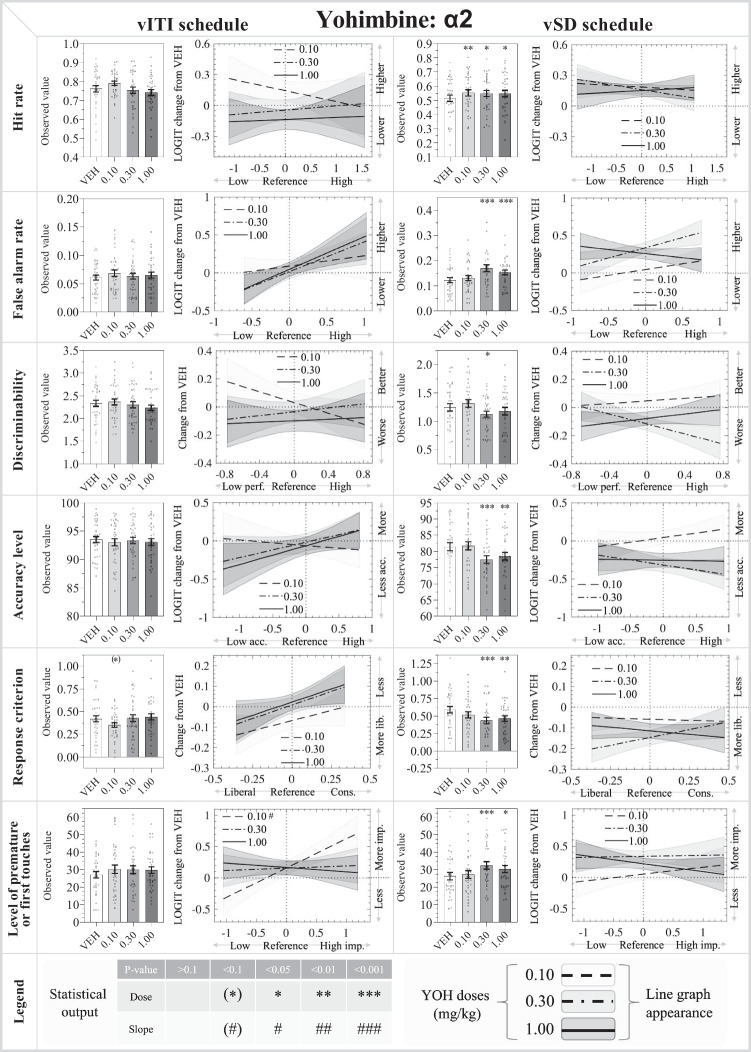
Results from the α_2_ adrenoceptor antagonist: yohimbine (YOH: 0.10, 0.30, 1.00 mg/kg) in the rodent continuous performance test. The left- and right-hand sides show the data from the vITI and vSD schedules, respectively. The bar charts depict the observed data on the original scale, while the line charts depict the modelled reference-dependent effects as analysed data with the appropriate transformations. The line chart y-axes denote changes relative to the within-subject VEH measurement within the Latin square design. The zero value on the x-axis indicates the mean of the reference values for the whole cohort. Significant reference-dependent treatment effects are reflected by line slopes that significantly differ from 0. The line graphs include a shaded standard error phase, depicting the modelled standard error of the dose at X=0, modified by the standard error of the slope towards the edges. Abbreviations: *vSD* variable stimulus duration, *vITI* variable intertrial interval, *VEH* vehicle, *FAR* false alarm rate, *imp.* impulsivity, *cons.* conservative. N for vITI: 36, vSD: 35

**Fig. 5:**
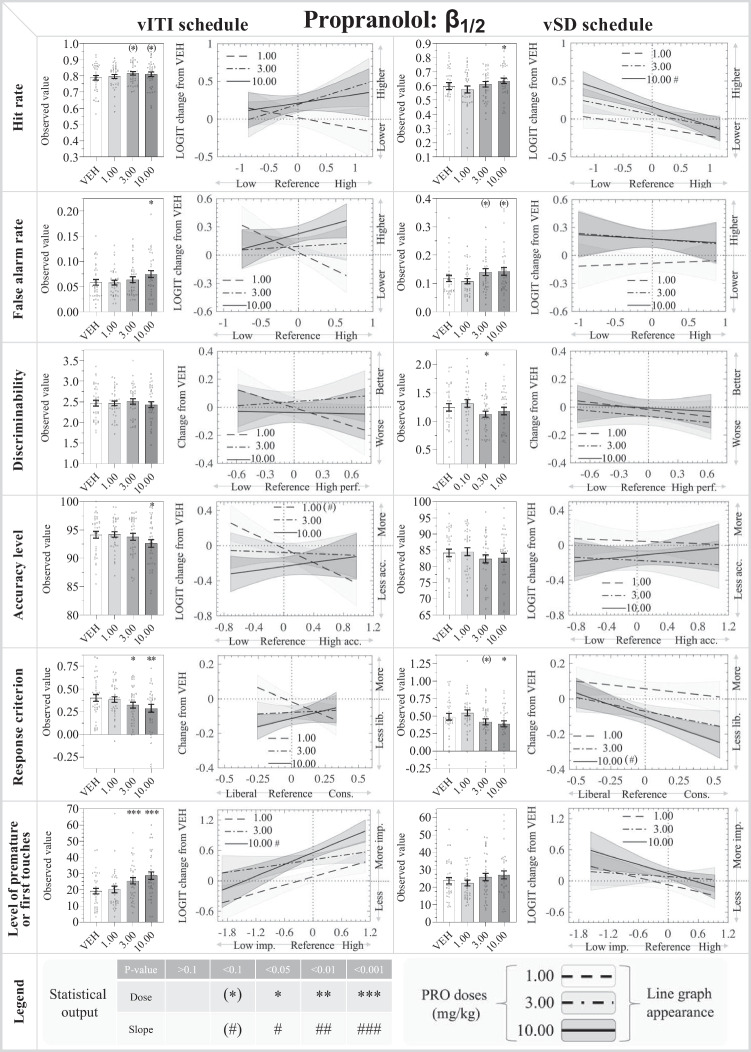
Results from the β_1/2_ adrenoceptor antagonist: propranolol (PRO: 1.00, 3.00, 10.00 mg/kg) in the rodent continuous performance test. The left- and right-hand sides show the data from the vITI and vSD schedules, respectively. The bar charts depict the observed data on the original scale, while the line charts depict the modelled reference-dependent effects as analysed data with the appropriate transformations. The line chart y-axes denote changes relative to the within-subject VEH measurement within the Latin square design. The zero value on the x-axis indicates the mean of the reference values for the whole cohort. Significant reference-dependent treatment effects are reflected by line slopes that significantly differ from 0. The line graphs include a shaded standard error phase, depicting the modelled standard error of the dose at X=0, modified by the standard error of the slope towards the edges. Abbreviations: *vSD* variable stimulus duration, *vITI* variable intertrial interval, *VEH* vehicle, *FAR* false alarm rate, *imp.* impulsivity, *cons.* conservative. N for vITI: 36, vSD: 35

**Fig. 6:**
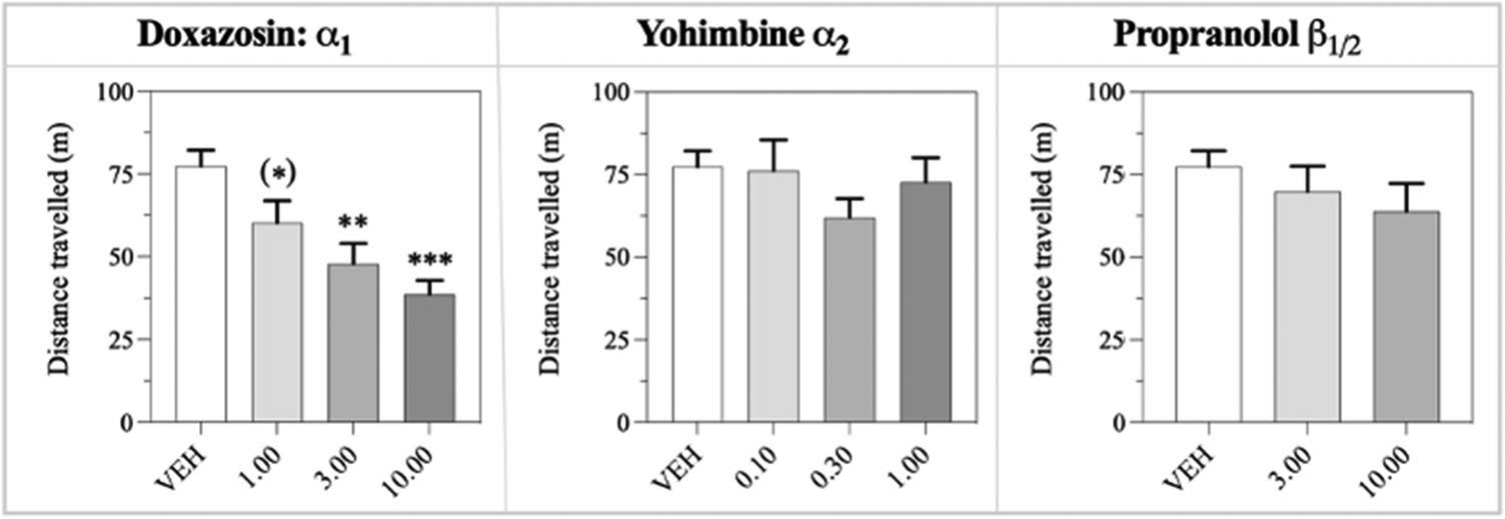
Total distance travelled in the locomotor assay, analysed through one-way ANOVA with multiple comparisons to the vehicle for the individual doses (Dunnett’s). The data was log-transformed prior to analysis and depicted as the non-transformed values (mean ± S.E.M.). The significance of individual doses is displayed, where trend values (0.05<P<0.1) are illustrated as (*), and significant values are illustrated as * / ** / *** for P < 0.05 / 0.01 / 0.001. N = 9–10 per dose.

The results from the drug experiments are presented in Figs. 3, 4, 5 in the order of testing (DOX, YOH, PRO). All results, both significant and non-significant, are compiled in Table 2 for the vITI data and Table 3 for vSD data. The effects of the drugs on rCPT response latencies are shown in Table 4, and the effects on LA are depicted in Fig. 6. A summary of all effects is presented in Table 5. There were statistically significant main and fixed effects of time and reference for most analyses. This shows that reference values are strong predictors of outcome values and confirms the importance of incorporating reference values in the statistical modelling. The significant main effects of time suggest a time-dependent drift for some parameters and confirm the importance of including time as a factor in the statistical modelling. The following sections describe the main effects on treatment, the post hoc analysis comparing the individual doses to the vehicle (including the fixed effects of dose and the dose:reference interactions), and the analysis of the response latencies. For brevity, only trend or significant main and fixed effects of dose, reference, and their interaction will be described in detail.

**Table 2:** Statistical output of the mixed effects model for the variable intertrial interval (vITI) schedule. The results were analysed in a repeated measures mixed effects model, using MATLAB version R2020b. The output is separated into the main effects from the model and those of the post hoc fixed effects comparisons to the vehicle. We examined doxazosin (1, 3, 10 mg/kg), yohimbine (0.1, 0.3, 1.0 mg/kg), and propranolol (1, 3, 10 mg/kg). Low, medium, and high refer to the relative concentrations of the drug doses. Significant effects (P<0.05) and trend-effects (0.05<P<0.1) are highlighted with bold font

Receptor, antagonist, and doses (mg/kg)		*α*_1_: Doxazosin 1.00, 3.00, 10.00	*α*_2_: Yohimbine 0.10, 0.30, 1.00	*β*_1/2_: Propranolol 1.00, 3.00, 10.00
Main effects				
Parameter	Variable	*F*_DF1,2_=Fstat, *P* value	*F*_DF1,2_=Fstat, *P* value	*F*_DF1,2_=Fstat, *P* value
Hit rate, HR	Time	***F***_**1, 138**_**=10.39,** ***P*<0.01**	***F***_**1, 138**_**=5.66,** ***P*<0.05**	*F*_1, 138_=1.36, *P*=0.246
	Dose	***F***_**3, 138**_**=8.22,** ***P*<0.001**	***F***_**3, 138**_**=2.91,** ***P*<0.05**	***F***_**3, 138**_**=2.17,** ***P*=0.095**
	Ref	***F***_**1, 138**_**=41.20,** ***P*<0.001**	***F***_**1, 138**_**=26.74,** ***P*<0.001**	***F***_**1, 138**_**=35.97,** ***P*<0.001**
	Dose:Ref	*F*_3, 135_=0.13, *P*=0.945	*F*_3, 135_=0.25, *P*=0.862	*F*_3, 135_=0.90, *P*=0.441
False alarm rate, FAR	Time	*F*_1, 138_=0.47, *P*=0.494	*F*_1, 138_=0.27, *P*=0.602	***F***_**1, 138**_**=4.03,** ***P*<0.05**
	Dose	***F***_**3, 138**_**=7.32,** ***P*<0.001**	*F*_3, 138_=0.33, *P*=0.800	***F***_**3, 138**_**=2.57,** ***P*=0.057**
	Ref	***F***_**1, 138**_**=26.52,** ***P*<0.001**	***F***_**1, 138**_**=14.60,** ***P*<0.001**	***F***_**1, 138**_**=18.55,** ***P*<0.001**
	Dose:Ref	*F*_3, 135_=0.43, *P*=0.733	*F*_3, 135_=1.07, *P*=0.366	*F*_3, 135_=2.06, *P*=0.108
Discriminability, d′	Time	***F***_**1, 138**_**=4.17,** ***P*<0.05**	***F***_**1, 138**_**=3.91,** ***P*<0.05**	*F*_1, 138_=0.49, *P*=0.486
	Dose	*F*_3, 138_=1.53, *P*=0.210	*F*_3, 138_=1.11, *P*=0.348	*F*_3, 138_=0.45, *P*=0.719
	Ref	***F***_**1, 138**_**=39.80,** ***P*<0.001**	***F***_**1, 138**_**=52.85,** ***P*<0.001**	***F***_**1, 138**_**=24.15,** ***P*<0.001**
	Dose:Ref	*F*_3, 135_=0.13, *P*=0.942	*F*_3, 135_=0.72, *P*=0.544	*F*_3, 135_=0.53, *P*=0.665
Accuracy level, %Acc	Time	*F*_1, 138_=0.01, *P*=0.927	*F*_1, 138_=0.67, *P*=0.415	*F*_1, 135_=2.18, *P*=0.142
	Dose	***F***_**3, 138**_**=3.29,** ***P*<0.05**	*F*_3, 138_=0.15, *P*=0.930	*F*_3, 135_=1.98, *P*=0.121
	Ref	***F***_**1, 138**_**=28.36,** ***P*<0.001**	***F***_**1, 138**_**=20.13,** ***P*<0.001**	***F***_**1, 135**_**=19.41,** ***P*<0.001**
	Dose:Ref	*F*_3, 135_=0.17, *P*=0.914	*F*_3, 135_=0.73, *P*=0.534	*F*_3, 135_=1.70, *P*=0.169
Response criterion, C	Time	***F***_**1, 138**_**=8.55,** ***P*<0.01**	*F*_1, 138_=1.25, *P*=0.266	***F***_**1, 138**_**=4.83,** ***P*<0.05**
	Dose	***F***_**3, 138**_**=14.05,** ***P*<0.001**	*F*_3, 138_=2.06, *P*=0.109	***F***_**3, 138**_**=3.75,** ***P*<0.05**
	Ref	***F***_**1, 138**_**=21.13,** ***P*<0.001**	*F*_1, 138_=0.50, *P*=0.686	***F***_**1, 138**_**=26.80,** ***P*<0.001**
	Dose:Ref	*F*_3, 135_=0.20, *P*=0.893	***F***_**3, 135**_**=17.21,** ***P*<0.001**	***F***_**3, 135**_**=3.85,** ***P*=0.052**
First touches level, %FiT	Time	*F*_1, 138_=0.25, *P*=0.621	*F*_1, 134_=0.97, *P*=0.410	***F***_**1, 135**_**=3.81,** ***P*<0.05**
	Dose	***F***_**3, 138**_**=26.05,** ***P*<0.001**	***F***_**3, 134**_**=28.86,** ***P*<0.001**	***F***_**3, 135**_**=26.76,** ***P*<0.001**
	Ref	***F***_**1, 138**_**=29.71,** ***P*<0.001**	***F***_**1, 134**_**=2.58,** ***P*=0.057**	*F*_1, 135_=1.61, *P*=0.191
	Dose:Ref	*F*_3, 135_=0.46, *P*=0.708	*F*_3, 134_=0.85, *P*=0.359	***F***_**3, 135**_**=28.97,** ***P*<0.001**
Post hoc fixed effects comparisons to the vehicle condition				
Parameter	Variable	EST ± SE, *P* value	EST ± SE, *P* value	EST ± SE, *P* value
Hit rate, HR	Intercept	0.692 ± 0.102	1.237 ± 0.088	1.534 ± 0.096
	Low	−0.026 ± 0.115, *P*=0.823	0.144 ± 0.097, *P*=0.139	0.020 ± 0.108, *P*=0.852
	Med	−0.063 ± 0.115, *P*=0.583	−0.045 ± 0.097, *P*=0.643	**0.196** ***±*** **0.108,** ***P*=0.072**
	High	**−0.491** ***±*** **0.115,** ***P*<0.001**	−0.136 ± 0.097, *P*=0.163	**0.213** ***±*** **0.108,** ***P*=0.052**
	Low:Ref	0.012 ± 0.204, *P*=0.953	−0.106 ± 0.185, *P*=0.567	−0.147 ± 0.241, *P*=0.544
	Med:Ref	0.066 ± 0.204, *P*=0.747	0.041 ± 0.185, *P*=0.823	0.235 ± 0.242, *P*=0.332
	High:Ref	0.111 ± 0.204, *P*=0.588	0.019 ± 0.185, *P*=0.916	0.112 ± 0.241, *P*=0.644
False alarm rate FAR	Intercept	−2.802 ± 0.089	−2.588 ± 0.080	−2.621 ± 0.080
	Low	0.089 ± 0.089, *P*=0.319	0.089 ± 0.096, *P*=0.354	0.028 ± 0.089, *P*=0.757
	Med	**−0.159** ***±*** **0.089,** ***P*=0.077**	0.016 ± 0.096, *P*=0.866	0.094 ± 0.089, *P*=0.297
	High	**−0.296** ***±*** **0.089,** ***P*<0.01**	0.047 ± 0.096, *P*=0.628	**0.227** ***±*** **0.089,** ***P*<0.05**
	Low:Ref	−0.110 ± 0.247, *P*=0.658	0.136 ± 0.285, *P*=0.634	−0.382 ± 0.247, *P*=0.124
	Med:Ref	−0.270 ± 0.246, *P*=0.274	0.394 ± 0.284, *P*=0.167	0.050 ± 0.247, *P*=0.841
	High:Ref	−0.178 ± 0.246, *P*=0.471	0.431 ± 0.285, *P*=0.132	0.214 ± 0.246, *P*=0.387
Discriminability, d′	Intercept	2.099 ± 0.078	2.277 ± 0.062	2.452 ± 0.063
	Low	−0.067 ± 0.084, *P*=0.426	0.031 ± 0.074, *P*=0.676	−0.004 ± 0.069, *P*=0.959
	Med	0.059 ± 0.084, *P*=0.486	−0.034 ± 0.074, *P*=0.649	0.042 ± 0.069, *P*=0.546
	High	−0.108 ± 0.084, *P*=0.202	−0.098 ± 0.074, *P*=0.189	−0.038 ± 0.069, *P*=0.583
	Low:Ref	0.137 ± 0.223, *P*=0.541	−0.192 ± 0.193, *P*=0.322	−0.216 ± 0.228, *P*=0.346
	Med:Ref	0.049 ± 0.222, *P*=0.826	0.069 ± 0.194, *P*=0.721	0.050 ± 0.228, *P*=0.827
	High:Ref	0.075 ± 0.223, *P*=0.738	0.027 ± 0.194, *P*=0.887	−0.014 ± 0.228, *P*=0.950
Accuracy level, %Acc	Intercept	1.931 ± 0.125	1.762 ± 0.095	1.875 ± 0.095
	Low	−0.136 ± 0.130, *P*=0.297	−0.060 ± 0.115, *P*=0.601	−0.033 ± 0.106, *P*=0.759
	Med	0.184 ± 0.130, *P*=0.159	−0.018 ± 0.115, *P*=0.878	−0.079 ± 0.106, *P*=0.457
	High	**0.221** ***±*** **0.130,** ***P*=0.091**	−0.063 ± 0.115, *P*=0.582	**−0.238** ***±*** **0.106,** ***P*<0.05**
	Low:Ref	−0.013 ± 0.257, *P*=0.961	−0.072 ± 0.254, *P*=0.777	**−0.404** ***±*** **0.244,** ***P*=0.099**
	Med:Ref	−0.165 ± 0.256, *P*=0.521	0.200 ± 0.253, *P*=0.431	−0.031 ± 0.243, *P*=0.897
	High:Ref	−0.079 ± 0.256, *P*=0.758	0.245 ± 0.254, *P*=0.336	0.118 ± 0.243, *P*=0.630
Response criterion, C	Intercept	0.659 ± 0.042	0.438 ± 0.035	0.371 ± 0.035
	Low	−0.020 ± 0.044, *P*=0.658	**−0.068** ***±*** **0.040,** ***P*=0.088**	−0.017 ± 0.039, *P*=0.653
	Med	0.066 ± 0.044, *P*=0.140	0.008 ± 0.040, *P*=0.838	**−0.079** ***±*** **0.039,** ***P*<0.05**
	High	**0.238** ***±*** **0.044,** ***P*<0.001**	0.022 ± 0.040, *P*=0.575	**−0.114** ***±*** **0.039,** ***P*<0.01**
	Low:Ref	−0.136 ± 0.212, *P*=0.520	0.197 ± 0.249, *P*=0.430	−0.321 ± 0.238, *P*=0.180
	Med:Ref	−0.136 ± 0.213, *P*=0.525	0.264 ± 0.248, *P*=0.289	0.036 ± 0.238, *P*=0.881
	High:Ref	−0.046 ± 0.212, *P*=0.827	0.257 ± 0.248, *P*=0.303	0.188 ± 0.237, *P*=0.429
First touches level, %FiT	Intercept	−0.925 ± 0.119	−0.880 ± 0.103	−1.326 ± 0.103
	Low	−0.174 ± 0.123, *P*=0.159	0.157 ± 0.113, *P*=0.168	0.079 ± 0.115, *P*=0.494
	Med	**−0.392** ***±*** **0.123,** ***P*<0.01**	0.152 ± 0.113, *P*=0.180	**0.416** ***±*** **0.115,** ***P*<0.001**
	High	**−1.014** ***±*** **0.123,** ***P*<0.001**	0.164 ± 0.113, *P*=0.149	**0.557** ***±*** **0.115,** ***P*<0.001**
	Low:Ref	−0.066 ± 0.219, *P*=0.764	**0.468** ***±*** **0.217,** ***P*<0.05**	0.268 ± 0.170, *P*=0.116
	Med:Ref	−0.094 ± 0.219, *P*=0.669	0.037 ± 0.217, *P*=0.866	0.138 ± 0.169, *P*=0.416
	High:Ref	0.146 ± 0.219, *P*=0.507	−0.070 ± 0.219, *P*=0.751	**0.388** ***±*** **0.170,** ***P*<0.05**

Abbreviations: *DF* degrees of freedom, *Fstat* F statistic, *SE* standard error of estimate, Ref reference. N: 36

**Table 3:** Statistical output of the mixed effects model for the variable stimulus duration (vSD) schedule. The results were analysed in a repeated measurements mixed effects model, using MATLAB version R2020b. The output is separated into the main effects from the model and those of the post hoc fixed effects comparisons to the vehicle. We examined doxazosin (1.00, 3.00, 10.00 mg/kg), yohimbine (0.10, 0.30, 1.00 mg/kg), and propranolol (1.00, 3.00, 10.00 mg/kg). Low, medium, and high refer to the relative concentrations of the drug doses. Significant effects (P<0.05) and trend effects (0.05<P<0.1) are highlighted with bold font

Receptor, antagonist, and doses (mg/kg)		*α*_1_: Doxazosin 1.00, 3.00, 10.00	*α*_2_: Yohimbine 0.10, 0.30, 1.00	*β*_1/2_: Propranolol 1.00, 3.00, 10.00
Main effects				
Parameter	Variable	*F*_DF1,2_=Fstat, *P* value	*F*_DF1,2_=Fstat, *P* value	*F*_DF1,2_=Fstat, *P* value
Hit rate, HR	Time	***F***_**1, 138**_**=30.37,** ***P*<0.001**	*F*_1, 132_=0.28, *P*=0.595	*F*_1, 131_=0.17, *P*=0.681
	Dose	***F***_**3, 138**_**=12.26,** ***P*<0.001**	***F***_**3, 132**_**=3.62,** ***P*<0.05**	***F***_**3, 131**_**=4.20,** ***P*<0.01**
	Ref	***F***_**1, 138**_**=65.30,** ***P*<0.001**	***F***_**1, 132**_**=99.99,** ***P*<0.001**	***F***_**1, 131**_**=91.34,** ***P*<0.001**
	Dose:Ref	*F*_3, 135_=0.96, *P*=0.414	*F*_3, 129_=0.38, *P*=0.768	*F*_3, 131_=1.59, *P*=0.194
False alarm rate, FAR	Time	***F***_**1, 138**_**=4.13,** ***P*<0.05**	***F***_**1, 132**_**=12.78,** ***P*<0.001**	***F***_**1, 134**_**=8.48,** ***P*<0.01**
	Dose	***F***_**3, 138**_**=15.09,** ***P*<0.001**	***F***_**3, 132**_**=9.77,** ***P*<0.001**	***F***_**3, 134**_**=4.17,** ***P*<0.01**
	Ref	***F***_**1, 138**_**=23.60,** ***P*<0.001**	***F***_**1, 132**_**=20.42,** ***P*<0.001**	***F***_**1, 134**_**=26.81,** ***P*<0.001**
	Dose:Ref	*F*_3, 135_=0.56, *P*=0.642	*F*_3, 129_=1.44, *P*=0.233	*F*_3, 131_=0.07, *P*=0.977
Discriminability, d′	Time	***F***_**1, 138**_**=6.01,** ***P*<0.05**	***F***_**1, 132**_**=7.64,** ***P*<0.01**	***F***_**1, 134**_**=4.17,** ***P*<0.05**
	Dose	***F***_**3, 138**_**=2.26,** ***P*=0.084**	***F***_**3, 132**_**=4.88,** ***P*<0.01**	*F*_3, 134_=0.52, *P*=0.668
	Ref	***F***_**1, 138**_**=50.86,** ***P*<0.001**	***F***_**1, 132**_**=41.22,** ***P*<0.001**	***F***_**1, 134**_**=97.73,** ***P*<0.001**
	Dose:Ref	*F*_3, 135_=0.69, *P*=0.559	*F*_3, 129_=1.58, *P*=0.197	*F*_3, 131_=0.11, *P*=0.955
Accuracy level, %Acc	Time	*F*_1, 138_=0.00, *P*=0.982	***F***_**1, 132**_**=11.63,** ***P*<0.001**	***F***_**1, 134**_**=8.08,** ***P*<0.01**
	Dose	***F***_**3, 138**_**=8.70,** ***P*<0.001**	***F***_**3, 132**_**=7.92,** ***P*<0.001**	*F*_3, 134_=1.69, *P*=0.172
	Ref	***F***_**1, 138**_**=30.80,** ***P*<0.001**	***F***_**1, 132**_**=35.80,** ***P*<0.001**	***F***_**1, 134**_**=50.39,** ***P*<0.001**
	Dose:Ref	*F*_3, 135_=0.85, *P*=0.471	*F*_3, 129_=0.58, *P*=0.627	*F*_3, 131_=0.13P=0.942
Response criterion, C	Time	***F***_**1, 135**_**=15.97,** ***P*<0.001**	***F***_**1, 132**_**=6.10,** ***P*<0.05**	***F***_**1, 131**_**=4.31,** ***P*<0.05**
	Dose	***F***_**3, 135**_**=18.52,** ***P*<0.001**	***F***_**3, 132**_**=6.49,** ***P*<0.001**	***F***_**3, 131**_**=6.18,** ***P*<0.001**
	Ref	***F***_**1, 135**_**=40.53,** ***P*<0.001**	***F***_**1, 132**_**=18.59,** ***P*<0.001**	***F***_**1, 131**_**=42.61,** ***P*<0.001**
	Dose:Ref	*F*_3, 135_=1.94, *P*=0.126	*F*_3, 129_=0.83, *P*=0.477	*F*_3, 131_=1.26, *P*=0.291
Premature response level, %PR	Time	*F*_1, 138_=0.72, *P*=0.398	***F***_**1, 132**_**=32.17 ,*P*<0.001**	***F***_**1, 134**_**=11.85,** ***P*<0.001**
	Dose	***F***_**3, 138**_**=11.37,** ***P*<0.001**	***F***_**3, 132**_**=5.99,** ***P*<0.001**	*F*_3, 134_=1.68, *P*=0.175
	Ref	***F***_**1, 138**_**=45.21,** ***P*<0.001**	***F***_**1, 132**_**=48.51,** ***P*<0.001**	***F***_**1, 134**_**=26.25,** ***P*<0.001**
	Dose:Ref	*F*_3, 135_=0.65, *P*=0.584	*F*_3, 129_=0.50, *P*=0.684	*F*_3, 131_=0.72, *P*=0.543
Post hoc fixed effects comparisons to the vehicle condition				
Parameter	Variable	EST ± SE, *P* value	EST ± SE, *P* value	EST ± SE, *P* value
Hit rate, HR	Intercept	−0.447 ± 0.076	0.068 ± 0.064	0.436 ± 0.074
	Low	0.071 ± 0.074, *P*=0.335	**0.185 ± 0.063,** ***P*<0.01**	−0.108 ± 0.078, *P*=0.167
	Med	**−0.126 ± 0.074,** ***P*=0.089**	**0.159 ± 0.063,** ***P*<0.05**	0.060 ± 0.078, *P*=0.438
	High	**−0.344 ± 0.074,** ***P*<0.001**	**0.151 ± 0.062,** ***P*<0.05**	**0.161 ± 0.078,** ***P*<0.05**
	Low:Ref	−0.127 ± 0.137, *P*=0.356	−0.028 ± 0.104, *P*=0.785	−0.119 ± 0.117, *P*=0.309
	Med:Ref	−0.222 ± 0.137, *P*=0.108	−0.079 ± 0.103, *P*=0.447	−0.152 ± 0.117, *P*=0.195
	High:Ref	−0.171 ± 0.137, *P*=0.214	0.027 ± 0.104, *P*=0.792	**−0.254 ± 0.118,** ***P*<0.05**
False alarm rate, FAR	Intercept	−2.005 ± 0.098	−1.827 ± 0.078	−1.904 ± 0.088
	Low	−0.016 ± 0.099, *P*=0.869	0.042 ± 0.074, *P*=0.574	−0.084 ± 0.091, *P*=0.357
	Med	**−0.270 ± 0.099,** ***P*<0.01**	**0.336 ± 0.074,** ***P*<0.001**	**0.178 ± 0.091,** ***P*=0.053**
	High	**−0.582 ± 0.099,** ***P*<0.001**	**0.256 ± 0.073,** ***P*<0.001**	**0.178 ± 0.091,** ***P*=0.053**
	Low:Ref	−0.294 ± 0.257, *P*=0.254	0.154 ± 0.196, *P*=0.432	0.031 ± 0.225, *P*=0.892
	Med:Ref	−0.279 ± 0.257, *P*=0.278	0.273 ± 0.191, *P*=0.154	−0.059 ± 0.226, *P*=0.795
	High:Ref	−0.217 ± 0.257, *P*=0.399	−0.110 ± 0.188, *P*=0.561	−0.045 ± 0.225, *P*=0.841
Discriminability, d′	Intercept	0.963 ± 0.052	1.198 ± 0.049	1.453 ± 0.055
	Low	0.055 ± 0.053, *P*=0.306	0.048 ± 0.047, *P*=0.309	−0.016 ± 0.060, *P*=0.786
	Med	0.080 ± 0.053, *P*=0.137	**−0.116 ± 0.047,** ***P*<0.05**	−0.067 ± 0.060, *P*=0.266
	High	**0.136 ± 0.053,** ***P*<0.05**	−0.077 ± 0.047, *P*=0.100	−0.005 ± 0.060, *P*=0.929
	Low:Ref	0.135 ± 0.196, *P*=0.493	0.047 ± 0.133, *P*=0.725	−0.084 ± 0.161, *P*=0.601
	Med:Ref	0.272 ± 0.197, *P*=0.169	−0.177 ± 0.132, *P*=0.183	−0.070 ± 0.161, *P*=0.666
	High:Ref	0.199 ± 0.196, *P*=0.313	0.079 ± 0.134, *P*=0.556	−0.038 ± 0.162, *P*=0.813
Accuracy level, %Acc	Intercept	0.287 ± 0.122	0.402 ± 0.092	0.688 ± 0.105
	Low	0.082 ± 0.129, *P*=0.527	0.044 ± 0.090, *P*=0.623	0.048 ± 0.112, *P*=0.670
	Med	**0.271 ± 0.129,** ***P*<0.05**	**−0.313 ± 0.090,** ***P*<0.001**	−0.174 ± 0.112, *P*=0.122
	High	**0.605 ± 0.129,** ***P*<0.001**	**−0.256 ± 0.089,** ***P*<0.01**	−0.119 ± 0.112, *P*=0.288
	Low:Ref	0.111 ± 0.302, *P*=0.714	0.120 ± 0.189, *P*=0.528	−0.036 ± 0.230, *P*=0.876
	Med:Ref	0.404 ± 0.303, *P*=0.184	−0.130 ± 0.189, *P*=0.492	−0.044 ± 0.230, *P*=0.848
	High:Ref	0.368 ± 0.302, *P*=0.225	−0.014 ± 0.190, *P*=0.943	0.084 ± 0.230, *P*=0.715
Response criterion, C	Intercept	0.754 ± 0.045	0.541 ± 0.040	0.456 ± 0.039
	Low	−0.016 ± 0.045, *P*=0.714	−0.057 ± 0.036, *P*=0.116	0.057 ± 0.041, *P*=0.162
	Med	**0.118 ± 0.045,** ***P*<0.01**	**−0.146 ± 0.036,** ***P*<0.001**	**−0.070 ± 0.041,** ***P*=0.085**
	High	**0.277 ± 0.045,** ***P*<0.001**	**−0.114 ± 0.035,** ***P*<0.01**	**−0.102 ± 0.041,** ***P*<0.05**
	Low:Ref	−0.268 ± 0.176, *P*=0.130	−0.022 ± 0.145, *P*=0.877	−0.085 ± 0.144, *P*=0.557
	Med:Ref	**−0.408 ± 0.176,** ***P*<0.05**	0.151 ± 0.141, *P*=0.286	−0.158 ± 0.145, *P*=0.277
	High:Ref	**−0.297 ± 0.176,** ***P*=0.094**	−0.069 ± 0.139, *P*=0.622	**−0.271 ± 0.145,** ***P*=0.064**
Premature response level, %PR	Intercept	−0.989 ± 0.110	−0.862 ± 0.087	−1.062 ± 0.100
	Low	−0.021 ± 0.119, *P*=0.859	0.048 ± 0.090, *P*=0.595	−0.074 ± 0.105, *P*=0.482
	Med	**−0.350 ± 0.119,** ***P*<0.01**	**0.337 ± 0.090,** ***P*<0.001**	0.082 ± 0.105, *P*=0.434
	High	**−0.593 ± 0.119,** ***P*<0.001**	**0.221 ± 0.089,** ***P*<0.05**	0.146 ± 0.105, *P*=0.166
	Low:Ref	−0.313 ± 0.240, *P*=0.195	0.110 ± 0.198, *P*=0.578	−0.226 ± 0.223, *P*=0.311
	Med:Ref	−0.239 ± 0.240, *P*=0.321	0.015 ± 0.199, *P*=0.938	−0.063 ± 0.223, *P*=0.778
	High:Ref	−0.123 ± 0.240, *P*=0.608	−0.129 ± 0.192, *P*=0.504	−0.283 ± 0.223, *P*=0.207

Abbreviations: *DF* degrees of freedom, *Fstat* F statistic, *SE* standard error of estimate, *Ref *reference. N: vSD DOX: 36, vSD YOH and PRO: 35

**Table 4:** Overview of treatment effects on response latencies within the rCPT schedules. The results were analysed in a one-way analysis of variance with multiple comparisons to the vehicle (Dunnett), using Prism 9. Note, the reward collection latency values do not include SEM, as the means were log-transformed for the analysis and then back transformed for this table. Significant effects (P<0.05) and trend effects (0.05<P<0.1) are highlighted with bold font

Drug	Latency (s)	Vehicle	Low dose	Medium dose	High dose
		Mean ± SEM	Mean ± SEM, *P* value	Mean ± SEM, *P* value	Mean ± SEM, *P* value
Variable intertrial interval schedule					
DOX	Correct	0.990 ± 0.029	1.000 ± 0.028, *P*=0.943	1.038 ± 0.032, *P*=0.181	**1.127 ± 0.033,** ***P*<0.001**
	Incorrect	0.711 ± 0.054	**0.891 ± 0.063,** ***P*=0.050**	**0.946 ± 0.075,** ***P*<0.05**	0.811 ± 0.084, *P*=0.645
	Collection	1.148	**1.216,** ***P*<0.05**	**1.331,** ***P*<0.001**	**1.489,** ***P*<0.001**
YOH	Correct	0.945 ± 0.022	0.926 ± 0.027, *P*=0.690	0.984 ± 0.024, *P*=0.158	0.964 ± 0.025, *P*=0.713
	Incorrect	0.917 ± 0.057	0.838 ± 0.044, *P*=0.497	0.887 ± 0.059, *P*=0.958	0.896 ± 0.067, *P*=0.990
	Collection	1.066	1.070, *P*=0.977	**1.028,** ***P*<0.05**	**1.028,** ***P*<0.01**
PRO	Correct	0.993 ± 0.028	0.977 ± 0.028, *P*=0.870	0.953 ± 0.023, *P*=0.193	**0.905 ± 0.027,** ***P*<0.001**
	Incorrect	1.006 ± 0.053	0.952 ± 0.044, *P*=0.770	1.001 ± 0.062, *P*>0.999	0.932 ± 0.049, *P*=0.670
	Collection	1.057	**1.103,** ***P*<0.01**	**1.106,** ***P*<0.05**	**1.186,** ***P*<0.001**
Variable stimulus duration schedule					
DOX	Correct	0.731 ± 0.022	0.755 ± 0.027, *P*=0.613	**0.786 ± 0.027,** ***P*<0.05**	**0.816 ± 0.028,** ***P*<0.01**
	Incorrect	1.155 ± 0.034	1.178 ± 0.037, *P*=0.948	1.126 ± 0.032, *P*=0.869	1.195 ± 0.053, *P*=0.854
	Collection	1.135	**1.175,** ***P*<0.05**	**1.325,** ***P*<0.001**	**1.450,** ***P*<0.001**
YOH	Correct	0.690 ± 0.026	0.670 ± 0.016, *P*=0.661	0.663 ± 0.018, *P*=0.401	0.664 ± 0.019, *P*=0.502
	Incorrect	1.069 ± 0.032	1.045 ± 0.024, *P*=0.887	1.073 ± 0.028, *P*>0.999	1.080 ± 0.026, *P*=0.984
	Collection	1.112	1.090, *P*=0.340	**1.072,** ***P*<0.05**	**1.073,** ***P*<0.05**
PRO	Correct	0.677 ± 0.016	0.697 ± 0.019, *P*=0.482	0.695 ± 0.019, *P*=0.545	0.668 ± 0.014, *P*=0.822
	Incorrect	1.045 ± 0.035	1.060 ± 0.026, *P*=0.972	1.072 ± 0.025, *P*=0.820	1.052 ± 0.030, *P*=0.995
	Collection	1.101	**1.163,** ***P*<0.001**	**1.191,** ***P*<0.001**	**1.232,** ***P*<0.001**

DOX doses: 1.00, 3.00, 10.00 mg/kg. YOH doses: 0.10, 0.30, 1.00 mg/kg. PRO doses: 1.00, 3.00, 10.00 mg/kg

The data were analysed in a repeated measurement mixed-effects model, with the treatments as fixed effects, while the subjects and residual were random effects.

The results from the post-hoc multiple comparison (Dunnett’s) are presented.

The reward collection latency data was log-transformed to comply with the analysis assumptions, following which the means were back-transformed for this Table.

Significant results (P<0.05 and below) and trend effects are highlighted in bold (P<0.1).

Abbreviations: *rCPT* rodent continuous performance test, *SEM* standard error of the mean, *P* P value, *DOX* doxazosin, *YOH* yohimbine, *PRO* propranolol. N: vITI: 36, vSD DOX: 36, vSD YOH and PRO: 35

**Table 5:** *Overview of findings in the rCPT vSD and vITI schedules, and in the locomotor assay*

Drug	Dose (mg/kg)	Probe	HR	FAR	d’	%Acc	C	%FiT or %PR	S+ Lat.	S- Lat.	Col. Lat.	LA
DOX *α*_1_	1.00	vITI	=	=	=	=	=	=	=	(↑)	↑	(↓)
		vSD	=	=	=	=	=	=	=	=	↑	
	3.00	vITI	=	(↓)	=	=	=	↓	=	↑	↑	↓
		vSD	(↓)	↓	=	↑	↑L	↓	↑	=	↑	
	10.00	vITI	↓	↓	=	(↑)	↑	↓	↑	=	↑	↓
		vSD	↓	↓	↑	↑	↑(LH)	↓	↑	=	↑	
YOH *α*_2_	0.10	vITI	=	=	=	=	(↓)	↑H	=	=	=	=
		vSD	↑	=	=	=	=	=	=	=	=	
	0.30	vITI	=	=	=	=	=	=	=	=	↓	=
		vSD	↑	↑	↓	↓	↓	↑	=	=	↓	
	1.00	vITI	=	=	=	=	=	=	=	=	↓	=
		vSD	↑	↑	=	↓	↓	↑	=	=	↓	
PRO *β*_1/2_	1.00	vITI	=	=	=	(↑L↓H)	=	=	=	=	↑	n.d.
		vSD	=	=	=	=	=	=	=	=	↑	
	3.00	vITI	(↑)	=	=	=	↓	↑	=	=	↑	=
		vSD	=	(↑)	=	=	(↓)	=	=	=	↑	
	10.00	vITI	(↑)	↑	=	↓	↓	↑HL	↓	=	↑	=
		vSD	↑L	(↑)	=	=	↓(H)	=	=	=	↑	

Analysis in repeated measurements mixed effect models, with post hoc fixed effects comparisons with vehicle

↑ and ↓ for increases and decreases, respectively, while = denotes non-significant effects

( ) For trend effects (*P*<0.1), either related to dose or dose:reference

*n*. *d*. for effects that were not determined

L for effects towards the low end on the given parameter’s scale

H for effects towards the high end on the given parameter’s scale

LH for effects in the same direction on both ends of the scale, but disproportionally affecting the first letter more

No letter for uniform effects on the given parameters scale, where the line graph slope is non-significant

Abbreviations: *rCPT* rodent continuous performance task, *vSD* variable stimulus duration schedule, *vITI* variable intertrial interval schedule, *HR* hit rate, *FAR* false alarm rate, *d′* discriminability, *%Acc* accuracy level, *C* response criterion, *%PR* premature response level, *%FiT* first touches level, *Lat* latency, *Col.* collect. N: vITI: 36, vSD DOX: 36, vSD YOH and PRO: 35

### Variable intertrial interval schedule

#### Doxazosin

*Main effects:* There was a significant main effect of treatment on HR (*F*_3,138_ = 8.22; *P* <0.001), FAR (*F*_3,138_ = 7.32; *P*<0.001), %Acc (*F*_3,138_ = 3.29; *P*<0.05), C (*F*_3,138_ = 14.05; *P*<0.001), and %FiT (*F*_3,138_ = 26.05; *P*<0.001), but not on d′.

*Specific dose effects:* Post hoc analysis of the effect of each dose compared to the vehicle showed that HR was significantly reduced by 10.00 mg/kg DOX (*P*<0.001). FAR was significantly reduced by 10.00 mg/kg DOX (*P*<0.01), and there was a trend for the 3.00 mg/kg dose to decrease FAR (*P*=0.077). d′ was not significantly affected by DOX. There was a trend for the 10.00 mg/kg dose to increase %Acc (*P*=0.091). Response criterion, C, was significantly increased by 10.00 mg/kg DOX (*P*<0.001), causing a more conservative response strategy. %FiT was dose-dependently reduced by DOX, with significant effects for 3.00 (*P*<0.01) and 10.00 mg/kg (*P*<0.001). None of these effects were reference-dependent.

*Latency effects:* At 10.00 mg/kg, DOX increased the correct latency (*P*<0.001); 1.00 mg/kg trended to increase the incorrect latency (*P*=0.050), while 3.00 mg/kg significantly increased incorrect latency (*P*<0.05). All DOX doses slowed the reward collection responses (*P*<0.05 for 1.00 mg/kg, *P*<0.001 for 3.00 and 10.00 mg/kg).

#### Yohimbine

*Main effects:* There was a significant main effect of treatment on HR (F_3,138_ = 2.91; *P*<0.05), but no significant main effects on the other parameters.

*Specific dose effects:* HR, FAR, d′, and %Acc were not significantly affected by any of the YOH doses tested. The 1.00 mg/kg dose showed a trend effect to reduce C (*P*=0.088), i.e., causing a more liberal response strategy, and this was not reference-dependent. The 1.00 mg/kg dose did not have a significant overall dose effect on %FiT, but showed a significant dose:reference interaction, with a more pronounced increase in %FiT in mice with high reference %FiT values (*P*<0.05).

*Latency effects:* YOH did not significantly affect the correct or incorrect latency, while the 0.30 mg/kg (*P*<0.05) and 1.00 mg/kg doses (*P*<0.01) made reward collection responses faster.

#### Propranolol

*Main effects:* There was a significant main effect of treatment on C (*F*_3,138_ = 3.75; *P*<0.05) and %FiT (*F*_3,135_ = 26.76; *P*<0.001), and trend effects on HR (*F*_3,138_ = 2.17; *P*=0.095) and FAR (*F*_3, 138_ = 2.57; *P*=0.057), but no significant effects on d′ or %Acc.

*Specific dose effects:* The 3.00 (*P*=0.072) and 10.00 mg/kg doses (*P*=0.051) were associated with trend increases in HR. FAR was significantly increased by 10.00 mg/kg (*P*<0.01). None of these effects were reference-dependent. d′ was not significantly affected by the tested doses of PRO. %Acc was significantly reduced by 10.00 mg/kg PRO (*P*<0.05), while the 1.00 mg/kg dose showed a trend dose:reference interaction (*P*=0.099) to improve %Acc in low-%Acc mice and worsen %Acc in high-%Acc mice. C was significantly reduced by 3.00 (*P*<0.05) and 10.00 mg/kg (*P*<0.01) PRO, and these effects were not reference-dependent. %FiT was dose-dependently increased by PRO, with significant values for 3.00 and 10.00 mg/kg (*P*<0.001 both doses). The 10.00 mg/kg dose showed a significant dose:reference interaction (*P*<0.05) to increase %FiT more prominently in high-%FiT mice.

*Latency effects:* At 10.00 mg/kg, PRO reduced the correct latency (*P*<0.001), and none of the doses affected the incorrect latency. All PRO doses increased the reward collection latency (*P*<0.01 for 1.00 mg/kg, *P*<0.05 for 3.00 mg/kg, *P*<0.001 for 10.00 mg/kg).

### Variable stimulus duration schedule

#### Doxazosin

*Main effects:* There was a significant main effect of treatment on HR (*F*_3,138_ = 12.26; *P* <0.001), FAR (*F*_3,138_ = 15.09; *P*<0.001), %Acc (*F*_3,138_ = 8.70; *P*<0.01), C (*F*_3,135_ = 18.52; *P*<0.001), and %PR (*F*_3,135_ = 11.37; *P*<0.001), while there was only a trend on d′ (*F*_3,138_ = 2.46; *P*=0.084).

*Specific dose effects:* HR was significantly reduced by the 10.00 mg/kg dose (*P*<0.001) with a trend reduction observed for the 3.00 mg/kg dose (*P*=0.089). FAR was significantly reduced by 3.00 (*P*<0.01) and 10.00 mg/kg (*P*<0.001). d′ was significantly increased by the 10.00 mg/kg dose (*P*<0.05). %Acc was significantly increased by 3.00 mg/kg (*P*<0.05) and 10.00 mg/kg (*P*<0.001). These effects were not reference-dependent. C was significantly increased by 3.00 mg/kg (*P*<0.01) and 10.00 mg/kg (*P*<0.001). The 3.00 mg/kg (*P*<0.05) and 10.00 mg/kg (*P*=0.095) doses showed significant and trend dose:reference interactions, respectively. Both doses increased C most prominently in low-C mice, while the 10.00 mg/kg dose moderately increased C in high-C mice. %PR was dose-dependently decreased by DOX with significant values for 3.00 mg/kg (*P*<0.01) and 10.00 mg/kg (*P*<0.001), which were not reference-dependent.

*Latency effects:* DOX slowed the correct responses at 3.00 mg/kg (*P*<0.05) and 10.00 mg/kg (*P*<0.001), while none of the tested doses affected the incorrect latency. All doses slowed the reward collection latency (*P*<0.05 for 1.00 mg/kg, *P*<0.001 for 3.00 and 10.00 mg/kg).

#### Yohimbine

*Main effects:* There was a significant main effects of treatment on all parameters (HR: *F*_3,132_ = 3.62; *P*<0.05) (FAR: *F*_3,132_ = 9.77; *P*<0.001) (d′: *F*_3,132_ = 4.88; *P* <0.01) (%Acc: *F*_3,132_ = 7.92; *P*<0.001) (C: *F*_3,132_ = 6.49; *P*<0.001) (%PR: *F*_3,132_ = 5.99; *P*<0.001).

*Specific dose effects:* Post hoc analysis of YOH treatment generally showed peak effects around the low-medium doses, 0.10 and 0.30 mg/kg, with lower effects at the 1.00 mg/kg dose. HR was significantly increased by YOH, with the peak effect at 0.10 mg/kg (*P*<0.01) and more modest increases at 0.30 and 1.00 mg/kg (both *P*<0.05). FAR was significantly increased by 0.30 mg/kg (*P*<0.001) and 1.00 mg/kg (*P*<0.05). d′ was significantly reduced by 0.30 mg/kg (*P*<0.05), and %Acc was significantly reduced by 0.30 mg/kg (*P*<0.001) and 1.00 mg/kg (*P*<0.01), indicating impaired attention. C was significantly reduced by 0.30 mg/kg (*P*<0.001) and 1.00 mg/kg (*P*<0.01), indicating that these doses induced a more liberal response strategy. %PR was significantly increased by 0.30 mg/kg (*P*<0.001) and 1.00 mg/kg (*P*<0.05). None of these effects were reference-dependent.

*Latency effects:* YOH did not significantly affect the correct or incorrect latency, but 0.30 (*P*<0.05) and 1.00 mg/kg (*P*<0.01) made reward collection responses faster.

#### Propranolol

*Main effects:* There was a significant main effect of treatment on HR (*F*_3,131_ = 4.20; *P*<0.01), FAR (*F*_3,134_ = 4.17; *P*<0.01), and C (*F*_3,131_ = 6.18; *P*<0.001), but not on d′, %Acc, or %PR.

*Specific dose effects:* HR was significantly increased by 10.00 mg/kg (*P*<0.05), and at this dose, a significant dose:reference interaction was seen (*P*<0.05), increasing HR more prominently in low-HR mice. The 3.00 and 10.00 mg/kg doses trended to increase FAR (both *P*=0.053), and none of the effects were reference-dependent. d′ and %Acc were not significantly affected by the tested PRO doses. C was significantly reduced by 10.00 mg/kg PRO (*P*<0.05), with a trend reduction for the 3.00 mg/kg dose (*P*=0.085). The 10.00 mg/kg dose had a trend dose:reference interaction (*P*=0.064) to reduce C more prominently in high-C mice. The 10.00 mg/kg dose showed a trend increase in %PR (*P*=0.090), and this effect was not reference-dependent.

*Latency effects:* PRO did not significantly affect correct or incorrect latencies, but all doses slowed the reward collection latency (*P*<0.01 for 1.00 mg/kg, *P*<0.05 for 3.00 mg/kg, *P*<0.001 for 10.00 mg/kg).

### Locomotor activity

The summary of the total distance travelled is presented in Fig. 6 below with the statistical output in the following paragraphs.

*Doxazosin:* The one-way ANOVA showed a significant main effect of treatment on locomotor activity (*F*_3, 33_ = 9.50; *P*<0.001). Post hoc analysis comparing the doses to the vehicle (Dunnett’s) revealed a dose-dependent decrease in distance travelled (*P*=0.091 for 1.00 mg/kg, *P*<0.01 for 3.00 mg/kg, *P*<0.001 for 10.00 mg/kg).

*Yohimbine:* The analysis did not show a significant main effect of treatment on locomotor activity (*F*_3, 33_ = 0.95; *P*=0.430), and post hoc analysis showed no significant effect of any of the doses.

*Propranolol:* The analysis showed no significant main effect of treatment on locomotor activity (*F*_2, 24_ = 0.891; *P*=0.423). Post hoc analysis found no significant effect of the two doses (*P*=0.684 for 3.00 mg/kg, *P*=0.324 for 10.00 mg/kg) compared to the vehicle. Note, 1.00 mg/kg PRO was not included in the experiment, as the dose was associated with unlikely motor effects based on previous research (Rodriguez-Romaguera et al. 2009; Hecht et al. 2014) and would reduce the number of mice available for other dose analyses.

### Data summary

An overview of all data is provided below in Table 5.

## Discussion

To examine the role of NA in attention and impulsivity, we assessed the behavioural output of NA R antagonists in the rCPT vSD and vITI schedules, and the drug effects were related to the reference level of each subject. The following sections begins with a summary of our findings, proceeds to discuss the effects on attention and impulsivity separately, and concludes with effects on locomotion and response latencies. In addition to affecting measures of attention and impulsivity, some of the drug doses examined in this current paper and in the adjoining paper examining DA receptor antagonists (Klem et al. 2023) also showed effects on measures of motor activity and motivation (response and reward collection latencies). Both attention and impulsive responses are partly dependent on motivation, and, similar to other operant tasks, the rCPT relies on motor responses. Therefore, any treatment effects on locomotor activity and/or response/reward collection latencies should therefore be considered when interpreting treatment effects on measures of attention or impulsivity.

### α_1_ adrenoceptor antagonism improved attentional performance in the vSD schedule, reduced overall responding in both rCPT schedules, and reduced locomotor activity

DOX reduced responding and impulsivity in both schedules, as measured by an increase in C and a reductions in HR, FAR, %FiT, and %PR. The reduced responding may be confounded by or partly attributable to a general reduction in motor activity, as reflected by reduced locomotor activity and the longer response and reward collection latencies. In the vSD schedule, DOX caused a more marked decrease in FAR than in HR, which was reflected by improvements in both d′ and accuracy. By contrast, DOX caused a similar drop in both FAR and HR in the vITI schedule, and consequently no significant change in d′ or %Acc. While this may support that the vSD schedule is more sensitive to changes in attentional performance than the vITI schedule, it was not a consistent finding across all antagonist studies.

The role of α_1_ adrenoceptor in modulating cognition is complicated and task-dependent. Some studies suggest that high NA levels may excessively activate α_1_ adrenoceptors and impair performance by negatively skewing the signal-to-noise ratio and ‘overshooting’ arousal along the inverted U-shaped arousal-performance relationship (Ramos and Arnsten 2007; Arnsten 2011). Our present finding that α_1_ adrenoceptor antagonism improved d′ and %Acc in the vSD schedule is in line with such a hypothesis. Our observation that DOX reduced HR is also in agreement with a rat 5-CSRTT study, where α_1_ adrenoceptor antagonism increased omissions (Adams et al. 2017). Interestingly, another rat 5-CSRTT study reported that α_1_ adrenoceptor agonism increased omissions and response latencies (Pattij et al. 2012). Unlike our finding of increased %Acc following DOX treatment, agonism or antagonism did not affect accuracy in the rat 5-CSRTT studies (Pattij et al. 2012; Adams et al. 2017). α_1_ adrenoceptor antagonism has also been examined in rats in the stop-signal reaction time (SSRT) task, which includes both go and no-go trials and therefore has some similarity to the rCPT HR and FAR, respectively. In the rat SSRT, α_1_ adrenoceptor antagonism decreased go accuracy and increased mean reaction time, which are in line with our observed reductions in HR and longer response latencies, but their lack of effect on stop accuracy is in contrast to our marked decrease in FAR (Bari and Robbins 2013). These findings show that both insufficient and excessive α_1_ adrenoceptor transmission have been associated with a more conservative response style (i.e., fewer and slower responses). Low α_1_ adrenoceptor activity represents a state of drowsiness and low vigilance, and has been associated with reduced firing of locus coeruleus NA neurons as well as inhibition of locomotor activity (Stone et al. 2004; Lin et al. 2007). In the present study, this is also reflected by the dose-dependent inhibition of both locomotor activity and overall responsivity in the rCPT (reduced HR, FAR, %PR, and %FiT) following DOX treatment. Conversely, high α_1_ adrenoceptor activity represents a state of hypervigilance, as observed in states of fear or anxiety (Atzori et al., 2016), during which a conservative response style is also conceivable.

The role of α_1_ adrenoceptors appears to be task-dependent. Agonism of α_1_ adrenoceptors shows beneficial effects in tasks that require divided attention or a high level of vigilance (Arnsten et al. 1999; Birnbaum et al. 2004; Baldi and Bucherelli 2005; Ramos and Arnsten 2007; Berridge and Spencer 2016; Spencer and Berridge 2019). These complicated effects may involve the opposing actions of α_1_ adrenoceptors on PFC neuronal excitability, as the adrenoceptors facilitate both excitatory and inhibitory transmission in the PFC (Marek and Aghajanian 1999; Mitrano et al. 2012; Xing et al. 2016). In addition, antagonism of pre-synaptic α_1_ adrenoceptors decreases extracellular DA levels in the ventral tegmental area (VTA) and nucleus accumbens (NAc) (Grenhoff and Svensson 1989, 1993; Grenhoff et al. 1993; Mitrano et al. 2012; Verheij et al. 2013; Goertz et al. 2015; Velásquez-Martínez et al. 2015). We have previously shown that chemogenetic inhibition of VTA neurons reduced locomotor activity and general responding in the 5-CSRTT (Fitzpatrick et al. 2019). It is therefore possible that our observed reductions in both responding and locomotor activity could involve reduced mesocorticolimbic DA activity.

Our observed slower responses, more conservative responding (increased C), and decreased locomotor activity may be partly due to decreased arousal or peripheral effects. The authors of the SSRT study attributed their results to the mild sedative effects of α_1_ adrenoceptor antagonism (Bari and Robbins 2013). As a previous study found that 0.3–1.0 mg/g of DOX administered intraperitoneally had no effect on or slightly increased motor activity in rats (Haile et al. 2012), we did not expect locomotor-depressant effects of the lower DOX doses. The effects of DOX on rCPT measures could therefore be confounded by a hypotensive and/or locomotor-depressant effect.

### α_2_ adrenoceptor antagonism increased overall responding and impaired attentional performance in the rCPT vSD schedule

In the vSD schedule, we observed that YOH increased both HR and FAR, with a stronger effect on FAR, as reflected by a decrease in d′ and %Acc. YOH trended to decrease C in the vITI schedule and significantly decreased C in the vSD schedule. There was a reference-dependent increase in %FiT in the vITI schedule. Increased %PR was also found in the vSD schedule, but this was not reference-dependent. YOH caused slower reward collection responses in both schedules, but no significant effect on correct or incorrect response latencies. YOH mostly showed effects in the vSD schedule, and only limited effects in the vITI schedule, which lends some support to the prediction that the vSD schedule is more sensitive towards effects on attentional measures. However, the effects of YOH on impulsivity were similar in both schedules.

Studies on the roles of NA receptors attention regulation, and specifically in the inverted U-shaped relationship between arousal/vigilance and attentional performance, have suggested opposite roles α_1_ and α_2A_ adrenoceptors. Activation α_2A_ adrenoceptor increases the sensitivity to target cues (signal) more than to non-target cues (noise), and the resulting increase in signal-to-noise ratio is believed to underlie the attention-enhancing effect of the α_2A_ adrenoceptor agonist guanfacine (GUA), used in the treatment of ADHD (Ramos and Arnsten 2007; Arnsten 2011). Our results support the opposing roles of α_1_ and α_2_ adrenoceptors, since antagonism of α_1_ receptors reduced impulsivity, improved attention, and decreased responding, whereas antagonism of α_2_ receptors increased impulsivity, worsened attention, and increased responding. We generally observed a YOH-induced inverted U-shaped dose-response profile. We speculate that this profile related to predominant antagonism of the presynaptic autoreceptors at the low-to-moderate doses, while the high dose may engage the post-synaptic adrenoceptors to a larger extent. Our observed impairment of attentional performance complies with a rat 5-CSRTT study where YOH decreased accuracy (Barlow et al. 2018). However, while we found an increase in overall responding (including reduced omissions), the study by Barlow et al. found no significant effect on omissions. Another rat 5-CSRTT study reported that α_2_ adrenoceptor agonism increased omissions, in accordance with our findings, although they did not find any effect on accuracy (Pattij et al. 2012). When comparing the mouse rCPT and rat 5-CSRTT, it is important to consider the incorporation of S− in the rCPT, which complicates comparisons between the two tasks. Discrepancies may also be attributed to differences between behavioural responses of rats and mice. Our observed worsening of attentional performance in the vSD schedule following α_2_ antagonism is in line with our previous work, where GUA increased d′ for low-performing male mice in a rCPT vSD schedule (Caballero-Puntiverio et al. 2019), and where similar GUA doses did not affect accuracy in a 5-CSRTT vITI study (Fitzpatrick and Andreasen 2019). The different sensitivities of the two rCPT schedules to detect YOH-induced effects on attentional performance may reflect differences in vigilance (and hence, catecholamine levels) required for optimal performance. The curve for the more difficult vSD schedule would be shifted to the left of the curve for the vITI schedule (Yerkes and Dodson 1908; Gould and Krane 1992; Diamond et al. 2007; Coon and Mitterer 2012; Yoon 2013), and the vSD schedule would be more sensitive to changes in α_2_ adrenoceptor activity at lower doses. It is also possible that attentional performance as a function of α_2_ adrenoceptor activity does not follow an inverted U-shaped curve. We are not aware of any behavioural studies showing detrimental effects of excessive α_2_ adrenoceptor stimulation, and it is possible that such a relationship is best described as a sigmoidal curve.

Differences between the two rCPT schedules were also observed for the impulsivity parameters, with YOH causing a more pronounced increase in impulsivity in the vSD schedule. These effects are in line with a rat 5-CSRTT study, where YOH showed a similar profile of increasing %PR in an inverted U-shaped dose-response pattern (Barlow et al. 2018). The increased %PR is also accordance with a 5-CSRTT rat study where an α_2_ adrenoceptor agonist decreased %PR (Pattij et al. 2012). In a previous rCPT vSD study, we found a reference-dependent effect of α_2_ adrenoceptor agonism with GUA, decreasing %PR in high-%PR mice but increased %PR in low-%PR mice (Caballero-Puntiverio et al. 2019). In a 5-CSRTT vITI study, we found no effects of GUA on %PR (Fitzpatrick and Andreasen 2019). These partly discrepant results may reflect differences between rCPT and 5-SCRTT and/or show that the effects of antagonism are not necessarily opposite to those of agonism, as discussed earlier for α_1_ adrenoceptor effects. Most of the rodent literature describes YOH-induced increases in motoric impulsivity in a delayed memory task in humans also showed increased impulsive behaviour following YOH treatment (Swann et al. 2005). However, some studies in humans reported reductions in different impulsive choice paradigms (Schippers et al. 2016), and even decreased motor impulsivity despite increased arousal (Herman et al. 2019). This suggests that both hypo- and hyper-arousal can engender similar response strategies. Overall, the discrepancies in outcome on impulsivity following α_2_ adrenoceptor manipulation suggest that the outcome of α_2_ adrenoceptor manipulation depends on the task setup, the examined behavioural subtypes, and the applied dose range.

Our observed YOH-induced increases in responding were mirrored by shorter reward collection latencies in both schedules, while no effects were observed on the correct or incorrect latencies, nor did the antagonist affect locomotor activity. This indicates that the examined YOH dose range may have influences motivation, but not causes unspecific effects on motor responses.

### β_1/2_ adrenoceptor antagonism increases responding in both rCPT schedules, decreases accuracy in the vITI schedule, and did not affect locomotor activity

In our study, PRO trended to increase HR in the vITI schedule and significantly increased HR in the vSD schedule. FAR was significantly reduced in the vITI schedule, and a trend reduction seen was in the vSD schedule. PRO decreased %Acc in the vITI schedule only, and there was no significant effects on d′ in either schedule. The overall increased responding was reflected by a decrease in C in both schedules and a significantly increased %FiT in the vITI schedule. PRO caused faster correct responses in the vITI schedule, but slowed reward collection responses in both schedules. Locomotor activity was not affected. The effects of PRO were generally more pronounced in the vITI schedule, most notably the impulsivity parameters. The significant effects on accuracy in the vITI schedule but not in the vSD schedule challenges the prediction that the vSD schedule is more sensitive towards effects on attentional measures, which was otherwise indicated by both DOX and YOH.

Although β blockers are mostly associated with their utility in managing clinical hypertension, β receptors also contribute to central NA transmission (Milstein et al. 2010). CNS β adrenoceptors regulate working memory and attention, where high NA levels impair performance through low-affinity β_1_ adrenoceptors (Ramos and Arnsten 2007). However, in the present study, PRO reduced accuracy in the vITI schedule, suggesting that β_1/2_ adrenoceptor activity may contribute to attentional performance at endogenous NA levels during rCPT performance. Ramos and colleagues examined the effects of subtype-specific and mixed β_1/2_ adrenoceptor agents on working memory in a delayed alternation T-maze in young and aged rats and monkeys, showing that PFC β_1_ adrenoceptor activation impair performance, while PFC β_2_ adrenoceptors improve performance (Ramos et al. 2005, 2008). The opposite actions of the two subtypes may explain why intra-PFC of the non-selective β_1/2_ adrenoceptor antagonist PRO showed no effect on spatial working memory in monkeys (Li and Mei 1994). However, a rat 5-CSRTT study showed an overall similar effect of β_1_ and β_2_ adrenoceptor agonism on accuracy (Pattij et al. 2012), suggesting that attention may not be differentially regulated by the two subtypes. The results from the 5-CSRTT rat study examining agonism comply with our antagonist-induced worsening of attentional performance in the vITI schedule.

Our PRO-induced increased impulsivity and responding (reduced omissions in both schedules, and faster reward collection responding in the vITI schedule) are in line with the aforementioned rat 5-CSRTT study, where both subtype-specific and mixed β_1/2_ adrenoceptor agonism increased omissions and slowed correct responses, and both β_1/2_ and β_2_ adrenoceptor agonism reduced %PR (Pattij et al. 2012). However, our findings oppose those of a rat SSRT study, where PRO decreased go accuracy, translating to decreased rCPT HR, and where no effect was found on stop accuracy, translating to rCPT FAR (Bari and Robbins, 2013). The reported similarities and differences between the roles of β_1_ and β_2_ adrenoceptors warrant examination of subtype-specific agonism and antagonism in the rCPT.

## Conclusion

This study used antagonism of NA adrenoceptors to characterise the role of NA in rCPT behaviours related to attention and impulsivity. While the discussion centred on attention and impulsivity, the non-specific outcomes of the systemically administered antagonists suggest that additional behavioural domains were engaged, including those related to motivation and locomotion. We observed similar outcomes of antagonising α_2_ or β_1/2_ adrenoceptors, while α_1_ adrenoceptor antagonism generally showed the opposite profile. Our antagonist results suggest that endogenous NA exerts bidirectional regulation of the behaviours measured in the rCPT, increasing responding through α_1_ adrenoceptors and reducing responding through α_2_ and β_1/2_ adrenoceptors. Endogenous NA appear to improve discriminability through α_2_ adrenoceptors and worsen discriminability through α_1_ adrenoceptors. Further, endogenous NA may improve accuracy through α_2_ and, to a lesser degree, β_1/2_ adrenoceptor activity, and worsen accuracy via α_1_ adrenoceptors.

The results also suggest that endogenous NA increases impulsivity and hastens both correct and incorrect responses through α_1_ adrenoceptors, reduces impulsivity through α_2_ or β_1/2_ adrenoceptor activity, and slows correct responses via β_1/2_ adrenoceptors. Our results also suggest that endogenous NA hastens reward collection responses through α_1_ and β_1/2_ adrenoceptors and slows reward collection responses through α_2_ adrenoceptors. Finally, our results suggest that activity of α_1_, but not β_1/2_ or α_1_, adrenoceptors is important for normal exploratory locomotor activity. The impulsivity, the different types latencies, and exploratory locomotor activity reflect different motivational factors. It is uncertain to what extent such motivational factors influence our core readouts of d′, accuracy and %PR or %FiT.

A secondary aim of our studies was to examine whether the effects of NA adrenoceptor antagonism differed between the parallel rCPT vSD and vITI studies. Research suggests that vSD paradigms are more attention-demanding, and vITI paradigms could be more sensitive to changes in impulsivity. The suggested sensitivities of the two schedules were supported by the more pronounced effects of DOX and YOH on attentional measures in the vSD schedule, but conflicted with the stronger effects of PRO on these measures in the vITI schedule. Also, the YOH-induced effects on both attentional performance and impulsivity were much more prominent in the vSD schedule. Overall, the directions of effects were similar in both schedules. The disparate outcomes indicate that the schedules do indeed differ in their sensitivities towards detecting different behaviours, but such may relate to sensitivities towards different types of antagonism rather than the behaviours themselves.

### Supplementary information

ESM 1

## Data Availability

The data that support the findings of this study are available from the corresponding author, upon reasonable request.
