# Supplementary material for: Assessing attention and impulsivity in the variable stimulus duration and variable intertrial interval rodent continuous performance test schedules using noradrenaline receptor antagonists in female C57BL/6JRj mice
Source: Psychopharmacology (Berl). 2023 Jun 17;240(8):1629–50. doi: 10.1007/s00213-023-06385-9 (PMC10349758; doi:10.1007/s00213-023-06385-9)
Supplement: Supplementary file 1 — ESM 1 [file 213_2023_6385_MOESM1_ESM.docx]

**Supplementary Material**

The supplementary material includes the following sections:

- The logit transformation
- The FoT/FiT ratio
- Dose-finding pilot study
- Test-retest reliability

**The logit transformation**

Logit transformation is not applicable if an observation reaches the lower or upper limit, such as FAR values of 0 or HR values of 1, as the logit value would become infinite. To accommodate such observations in the logit transformation procedure, we therefore used *theoretical* limits that are slightly outside the *absolute* limits, when necessary. Based on a pooling of historical data we established specific criteria for each parameter in both the vSD and the vITI schedules. When clustering of the data was observed, this was incorporated in the definition of theoretical limits. The calculation of an upper limit is exemplified for %Acc in the vITI schedule:

1. Calculate the range between the lowest and highest observed two values: 100% – 72.50% = 27.5%
2. Define the upper 10 % of the range: here, 10% of the range equals 2.75%, i.e., the upper 10% of the range is 97.25%; 100%.
3. Of all observations, calculate the proportion of observations that are within 10% of the observed range: 357 observations out of the 1278 total observations = 27.93%
4. The calculated upper limit is defined as the highest observed value + 5% of the proportion calculated in (3.): 100.000 + (5% of 27.934 %) = 101.397
   1. The 5% of the theoretical maximum were an important addition to secure the added values were appropriate based on the range of the parameter, i.e., the value was 5% for percentage data (%Acc, %FiT, and %PR), and 0.05 % for ratio data (HR and FAR)

Table 6 contains relevant information pertaining the logit transformation.

**Table 6** *Information pertaining logit transformations. Compiled based on 22 drug study studies, 11 in each of the schedules, where the compiled drugs had opposing effects on all parameters. Abbreviations: vITI: variable intertrial interval, vSD: variable stimulus duration, HR: hit rate, FAR: false alarm rate, %Acc: accuracy, %FiT: first touches level, %PR: premature response level*

| Schedule | Variable | HR | FAR | %Acc | %FiT or %PR |
| --- | --- | --- | --- | --- | --- |
| vITI | Observed lowest; highest | 0.073; 0.977 | 0.007; 0.329 | 72.500; 100.000 | 0.000; 77.087 |
|  | Proportion of observations within 10% of lower/upper | Lower: 0.391%  Upper: 12.128% | Lower: 41.315%  Upper: 0.078% | Lower: 0.078%  Upper: 27.934% | Lower: 15.650%  Upper: 0.157% |
|  | Clustering | Lower: No  Upper: Yes | Lower: Yes  Upper: No | Lower: No  Upper: Yes | Lower: Yes  Upper: No |
|  | Limits | Lower: 0.000  Upper: 0.983 | Lower: -0.013  Upper: 1.000 | Lower: 50.000  Upper: 101.397 | Lower: -0.782  Upper: 100.000 |
| vSD | Observed lowest; highest | 0.034; 0.888 | 0.004; 0.556 | 54.483; 100.000 | 0.191; 63.465 |
|  | Proportion of observations within 10% of lower/upper | Lower: 1.343%  Upper: 2.923% | Lower: 32.622%  Upper: 0.079% | Lower: 0.237%  Upper: 8.610% | Lower: 13.507%  Upper: 1.501% |
|  | Clustering | Lower: No  Upper: No | Lower: Yes  Upper: No | Lower: No  Upper: Yes | Lower: Yes  Upper: No |
|  | Limits | Lower: 0.000  Upper: 1.000 | Lower: -0.013  Upper: 1.000 | Lower: 50.000  Upper: 100.430 | Lower: -0.484  Upper: 100.000 |

**The FoT/FiT ratio**

Our previous study modified the analysis protocol for a rCPT vITI schedule and led to the development of the %FiT and FoT/FiT parameters based on the seemingly unrelated nature of FiT and FoT responses (Prichardt et al. 2023). Our study showed the two parameters were uncorrelated and were differentially sensitive to catecholamine manipulations, as amphetamine reduced %FiT and increased the FoT/FiT ratio, while atomoxetine reduced %FiT but did not affect the FoT/FiT ratio (Prichardt et al. 2023). These results indicated the rCPT %FiT parameter provides a more sensitive measure of waiting impulsivity compared to rCPT %PR, but such remains to be validated. Furthermore, as FiTs in the 5-CSRTT trigger a timeout period, during which potential FoTs are not recorded, the rCPT %FiT parameter may more closely translate to %PR measured in the 5-CSRTT (Bari et al. 2008, Prichardt et al. 2023). In our current rCPT study, we included the FoT/FiT ratio results to support characterisation of the parameter but refrain from speculation concerning the nature of the behaviour.

The FoT/FiT ratio is calculated based on the following:

FoT/FiT = (Centre touches during 12s ITI restart loops within 0-0.5s) / (Initial centre touches during 12s ITI)

The FoT/FiT ratio was not restricted by ceiling effects, but still required transformation to comply with the model assumptions, hence the results were transformed using the natural logarithm (ln). The FoT/FiT ratio results are presented in Appendix Figure 7 and in Table 7. The following sections describe the main effects of treatment, the dose:reference interaction, and the post-hoc analysis of the individual doses for each antagonist on the FoT/FiT ratio in the vITI schedule. For brevity, only trend or significant main and fixed effects will be described in detail.

*Doxazosin:* There was a significant main effect of treatment on the FoT/FiT ratio (F_3,135_ = 3.56; P<0.05). Post-hoc analysis of the effect of each dose compared to the vehicle showed that the FoT/FiT ratio was significantly increased by 10.00 mg/kg DOX (P<0.01). The 10.00 mg/kg dose showed a significant dose:reference interaction (P<0.001), increasing the ratio more prominently in high FoT/FiT mice.

*Yohimbine:* The main effect of treatment was not significant for the FoT/FiT ratio. The post-hoc analysis did not show any significant effects of the individual doses, nor did the analysis reveal significant dose:reference interactions.

*Propranolol:* There was a trend main effect of treatment on the FoT/FiT ratio (F_3,135_ = 2.36; P=0.075). The post-hoc analysis showed 10.00 mg/kg PRO (P<0.05) significantly increased the FoT/FiT ratio, and this effect was not reference-dependent. The increases in both %FiT and the FoT/FiT ratio mean that mice initiated more first touches and even more frequent subsequent touches.

Our current study supports that FiTs and FoTs are distinct behaviours, as DOX showed opposing effects on %FiT and FoT/FiT, while PRO increased both parameters. The unrelated natures of %FiT and FoT/FiT ratio in our vITI schedule highlight the need to separate rCPT %PR into these two parameters, at least to provide a more sensitive measurement of waiting impulsivity as reflected by the %FiT parameter. While further pharmacological characterisation is required to ascertain the nature of the FoT/FiT ratio, our current findings indicate FoT behaviour is sensitive to noradrenergic manipulation, specifically through α_1_ or β_1/2_ adrenoceptor activity.

**Table 7** *Statistical output of the mixed effects model for following to first touches ratio (FoT/FiT) in the the variable intertrial interval (vITI) schedule. The results were analysed in a repeated measures mixed effects model, using MATLAB version R2020b. The output is separated into the main effects from the model and those of the post-hoc fixed effects comparisons to the vehicle. We examined doxazosin (1, 3, 10 mg/kg), yohimbine (0.1, 0.3, 1.0 mg/kg), and propranolol (1, 3, 10 mg/kg). Low, medium, and high refer to the relative concentrations of the drug doses. Significant effects (P<0.05) are highlighted with grey. Trend-effects (0.05<P<0.1) are highlighted with light grey. Abbreviations: DF: degrees of freedom, Fstat: F statistic, SE: standard error of estimate, Ref: reference. N: 36*

| Receptor, antagonist,  and doses (mg/kg) | | $\alpha_{1}$: Doxazosin  1.00, 3.00, 10.00 | $\alpha_{2}$: Yohimbine  0.10, 0.30, 1.00 | $\beta_{1/2}$: Propranolol  1.00, 3.00, 10.00 |
| --- | --- | --- | --- | --- |
| Main effects | | | | |
| Parameter | Variable | F_DF1,2_=Fstat, P-value | F_DF1,2_=Fstat, P-value | F_DF1,2_=Fstat, P-value |
| Following to first  touches ratio,  FoT/FiT | Time | F_1, 135_=0.35, P=0.553 | F_1, 137_=0.85, P=0.360 | F_1, 138_=10.74, P<0.001 |
|  | Dose | F_3, 135_=3.56, P<0.05 | F_3, 137_=1.53, P=0.210 | F_3, 138_=46.52, P<0.001 |
|  | Ref | F_1, 135_=93.36, P<0.001 | F_1, 137_=80.70, P<0.001 | F_1, 138_=1.95, P=0.125 |
|  | Dose:Ref | F_3, 135_=5.86, P<0.001 | F_3, 134_=0.70, P=0.556 | F_3, 135_=4.43, P<0.05 |
| Post-hoc fixed effects comparisons to the vehicle condition | | | | |
| Parameter | Variable | EST $\pm$ SE, P-value | EST $\pm$ SE, P-value | EST $\pm$ SE, P-value |
| Following to first  touches ratio,  FoT/FiT | Intercept | 1.226 $\pm$ 0.030 | 1.220 $\pm$ 0.048 | 1.139 $\pm$ 0.054 |
|  | Low | -0.003 $\pm$ 0.029, P=0.903 | 0.017 $\pm$ 0.049, P=0.734 | 0.05 $\pm$ 0.056, P=0.379 |
|  | Med | 0.041 $\pm$ 0.029, P=0.150 | -0.023 $\pm$ 0.049, P=0.637 | -0.011 $\pm$ 0.056, P=0.843 |
|  | High | 0.077 $\pm$ 0.029, P<0.01 | -0.081 $\pm$ 0.049, P=0.102 | 0.130 $\pm$ 0.056, P<0.05 |
|  | Low:Ref | 0.188 $\pm$ 0.126, P=0.138 | 0.011 $\pm$ 0.120, P=0.928 | 0.001 $\pm$ 0.145, P=0.992 |
|  | Med:Ref | 0.038 $\pm$ 0.127, P=0.764 | 0.089 $\pm$ 0.120, P=0.459 | -0.045 $\pm$ 0.145, P=0.754 |
|  | High:Ref | 0.475 $\pm$ 0.126, P<0.001 | -0.078 $\pm$ 0.120, P=0.516 | -0.104 $\pm$ 0.145, P=0.475 |


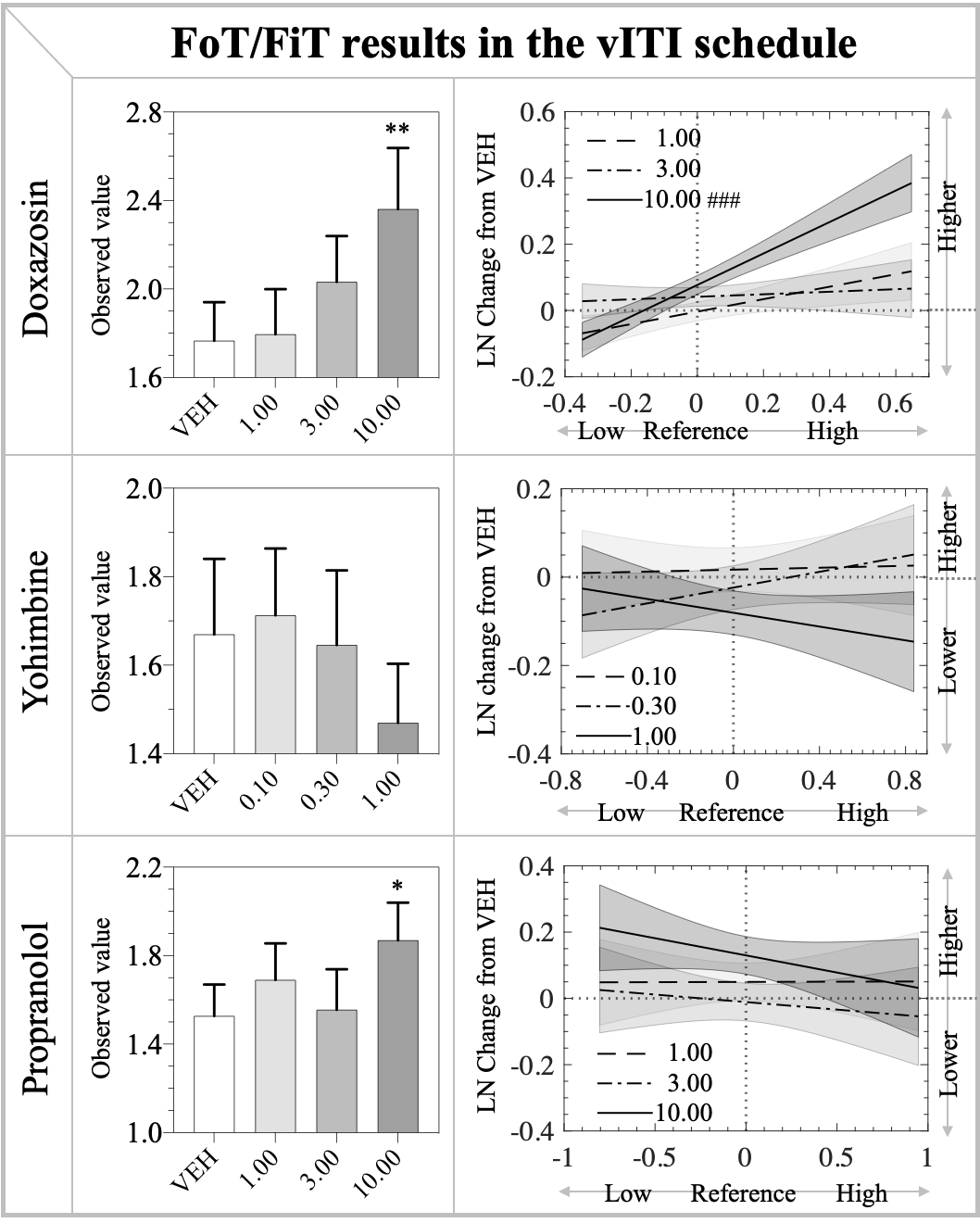


**Fig. 7** *Results from the a_1_ adrenoceptor antagonist: doxazosin (DOX: 1.00, 3.00, 10.00 mg/kg), a_2_ adrenoceptor antagonist: yohimbine (YOH: 0.10, 0.30, 1.00 mg/kg), and b_1/2_ adrenoceptor antagonist: propranolol (PRO: 1.00, 3.00, 10.00 mg/kg) in the rodent continuous performance test variable intertrial interval (vITI) schedule. The bar charts depict the observed data, while the line graphs depict the analysed data with the appropriate transformations. The line graph y-axes denote changes from the vehicle (VEH) measurement within the Latin square design. The line graph x-axes arrange the mice according to their average flanking vehicle measurements obtained outside of the Latin square design. Significant reference-dependent effects are shown as significant line graph slopes. The line graphs include a shaded standard error phase, depicting the standard error of the mean for the dose as the phase height at X=0, which is modified by the standard error of the slope towards the edges of the line. N: 36*

**Dose-finding pilot study**

The pilot doses were selected based on both motor and non-motor research, attempting to find doses affecting non-motor behaviours without non-specific motor effects. A pilot study was performed to find appropriate doses of the NA/DA R antagonists in the rCPT vSD schedule, using 19 male mice trained in the rCPT and habituated to the vSD schedule. The experimental design, drug preparation, and data analysis approach was the same as outlined in the main manuscript: individual Latin Square Designs for each drug flanked by reference measurements, modelled and analysed in line graphs using MATLAB. Brief descriptions of the outcomes are provided in the following, and an overview of the direction and significance of effects in provided in Table 8.

*DOX (0.50, 1.50, 4.50 mg/kg):* DOX doses were based on Haile et al., 2012. In the pilot experiment, DOX showed the most prominent effects for 4.50 mg/kg, while few effects were found for 0.50 mg/kg. As we assumed that we were below the peak level of effects, we decided to increase the dose range to 1.00, 3.00, and 10.00 mg/kg.

*YOH (0.35, 0.70, 1.40 mg/kg):* Previous studies have shown that YOH significantly increases impulsivity in an inverted U-shaped pattern (Mantsch et al., 2010, Barlow, Dalley and Pakcec, 2018; Mahoney et al., 2016). In the pilot experiment, the most prominent effects were observed for the lowest dose, 0.35 mg/kg YOH, while the effects tapered off at the higher doses. Therefore, we centred our new dose range around the lowest pilot dose: 0.10, 0.30, and 1.00 mg/kg.

*PRO (1.75, 3.50, 7.00 mg/kg):* Few effects were found for the medium and high dose of PRO, 3.50 and 7.00 mg/kg respectively, while low dose PRO, 1.75 mg/kg, showed no significant effects. To explore the impact of a higher dose, while staying below blood-concentrations with reported effects on blood pressure and locomotion (Mantsch et al., 2010), the new dose range was centred around the medium dose: 1.00, 3.00, 10.00 mg/kg.

*SCH (0.01, 0.03, 0.10 mg/kg):* SCH doses of 0.01-0.10 mg/kg were chosen for the pilot experiment based on previous behavioural studies (Arnsten and Dudley, 2005; Xueliang Fan and Hess, 2008). In the pilot experiment, the medium and higher SCH doses, 0.03 and 0.10 mg/kg respectively, showed very prominent effects, while few effects were found for low dose SCH, 0.01 mg/kg. However, 0.70 mg/kg SCH completely blocked activity as the mice appeared sedated. We therefore decided to lower the dose range to 0.01-0.04 mg/kg.

*RAC (0.07, 0.21, 0.70 mg/kg):* RAC doses were chosen based on previous *in vivo* binding studies and locomotion activity studies (Cumming et al., 2002; Xueliang Fan and Hess, 2008; Grimm et al., 2018). The higher doses of RAC, 0.21 and 0.70 mg/kg, showed prominent effects, while few significant effects were found for low dose RAC, 0.07 mg/kg. Therefore, we centred the new dose range around the low-medium doses: 0.03-0.30 mg/kg.

Overall, the directions of drug effects were similar for the male mice in the pilot study and in the female mice in the main study. This notably included the inverted U-shaped profile of yohimbine, and seemingly biphasic profile of raclopride.

**Table 8**: *Overview of the dose-finding pilot study results. Arrows show the direction of effects next to the significance. Values were considered significant for P<0.05*, P<0,01**, P<0.001***, while trend effects were described in parenthesis P<0.1(*). Non-significant effects were shown with dashes. Abbreviations: HR: hit rate, FAR: false alarm rate, d’: discriminability, C: response criterion, %PR: premature response level. N=19 male mice*

| **Drug** | **Doxazosin** | | | **Yohimbine** | | | **Propranolol** | | | **SCH23390** | | | **Raclopride** | | |
| --- | --- | --- | --- | --- | --- | --- | --- | --- | --- | --- | --- | --- | --- | --- | --- |
| **Pilot doses**  **(mg/kg)** | 0.50 | 1.50 | 4.50 | 0.35 | 0.70 | 1.40 | 1.75 | 3.50 | 7.00 | 0.01 | 0.03 | 0.10 | 0.07 | 0.21 | 0.70 |
| **HR** | - | - | - | ↑*** | ↑* | ↑* | - | - | - | - | ↓*** | ↓*** | - | ↓** | ↓*** |
| **FAR** | ↓* | ↓* | ↓*** | ↑** | - | - | - | ↑* | - | - | ↓*** | ↓*** | ↓* | ↓*** | ↓*** |
| **d’** | - | - | - | - | - | - | - | - | - | - | ↓(*) | ↓*** | ↑(*) | - | ↓(*) |
| **C** | ↑* | ↑* | ↑** | ↓*** | ↓* | ↓(*) | - | - | - | ↑(*) | ↑*** | ↑*** | - | ↑*** | ↑*** |
| **%PR** | - | - | ↓* | ↑*** | ↑(*) | ↑* | - | ↑** | ↑* | ↓* | ↓*** | ↓*** | ↓* | ↓*** | ↓*** |
| **Selected doses**  **(mg/kg)** | 1.00 | 3.00 | 10.00 | 0.10 | 0.30 | 1.00 | 1.00 | 3.00 | 10.00 | 0.01 | 0.02 | 0.04 | 0.03 | 0.10 | 0.30 |

**Test-retest reliability**

**The relationship between the reference values and the Latin Square Design values**

The statistical design relies on the relationship between the reference measurements obtained outside the Latin Square Design (LSD) and within the LSD. The statistical analysis includes P-values for reference that describe the extent to which the performance obtained outside the LSD contributes towards the performance obtained within the LSD. For example, a significant reference value for d’ generally informs that a mouse with a low average reference d’ will also have low d’ values within the LSD. The P-values for reference shown in Tables 2-3 were generally very significant, showing the strong relationship between performance inside and outside the LSD.

Given the nature of the assay and the experimental design, we anticipate increased performance (d’) over time. However, such changes were not an issue since these are accounted for in the statistical model with the time factor. Furthermore, the randomized LSD distributes any changes in time equally across all four doses. The P-values for time shown in Tables 2-3 were very significant, showing the importance of taking time into account. The changes in performance over time also signify the importance in measuring reference performance both prior to and after the LSD, rather than relying on a single reference measurement.

All mice ran the stage four (baseline) schedule on the day prior to each test day, corresponding to three-to-four days since the last test day. All mice were inspected for regular performance on these baseline days, ensuring a minimal carryover effect of treatment. Furthermore, the randomized LSD would distribute any carryover drug effect evenly across all doses, ensuring minimal interference in the interpretation of drug effects.

**The relationship between the reference values outside the Latin square design (LSD)**

We analysed the relationship between the six reference measurements in the vSD schedule for d’, C, and %PR using Pearson’s correlations in GraphPad Prism (version 9). Between each reference measurement, the drug LSD were performed, meaning each LSD is separated by around 3 weeks. We performed two separate analyses. The first analysis examined the absolute values obtained and the second analysis examined the relative performance hierarchies by ranking the 35 mice from worst-to-best for each reference measurement. The mean reference values and the hierarchy positions are shown in Table 9, and the outputs of the correlations analyses are shown in Table 10.

*Discriminability (d’):*  Overall, the reference d’-values were highly correlated. Two sets of reference values were not correlated: Ref1-Ref4, and Ref1-Ref5, while the correlation between the latter was borderline-significant in the hierarchy analysis. It is unsurprising that the first reference performance (Ref1) is less correlated with later performances, relative to the stronger correlations between subsequent reference values.

*Response criterion (C):* As for d’, the reference C-values were highly correlated with the exception for two reference sets: Ref1-Ref6 and Ref2-Ref6. However, the Ref-1-Ref-6 showed a trend correlation in the analysis of the absolute values, while the latter Ref2-Ref6 showed a trend in the hierarchy analysis. As for d’, it is unsurprising that the adjacent reference measurements all strongly correlated, while earlier reference measurements are less correlated with later values.

*Premature response level (%PR):* All adjacent %PR-values were correlated. Some sets of references were not significantly correlated: Ref1-Ref3, Ref1-Ref6, Ref 2-Ref6, while Ref4-Ref6 showed trend correlations in both the absolute and hierarchy analysis.

It was important for the statistical design that the adjacent reference values were highly correlated, as the analysis arranges the mice according to their average reference values across the line graph X-axis. If adjacent reference values were not correlated, it would signify that the positions on the line graph X-axes were arbitrary. Therefore, the strong statistical correlation between all adjacent reference values supports the statistical approach and reliability of performance across the 5-week testing period that includes the LSD and reference measurements. Overall, there was also a strong correlation between the non-adjacent references, while some did not show a significant correlation. The lack of significant relationship between a few of the non-adjacent references were not important for the statistical analysis but suggest that it may be inappropriate to conduct the individual LSDs over a longer period. For example, it might be inappropriate to have a design spanning >9 weeks, e.g., an LSD with sixteen doses and two flanking reference measurements.

**Table 9**: *Overview of the six reference values (ref1-6) for discriminability, response criterion, and premature response level for the 35 female mice in the rodent continuous performance test variable stimulus duration schedule. The values are presented as the absolute values and the relative rank within the hierarchy going from lowest-to-highest (1-35)*

| **Value** | **Absolute discriminability** | | | | | | **Hierarchy discriminability** | | | | | |
| --- | --- | --- | --- | --- | --- | --- | --- | --- | --- | --- | --- | --- |
| **ID** | **Ref1** | **Ref2** | **Ref3** | **Ref4** | **Ref5** | **Ref6** | **Ref1** | **Ref2** | **Ref3** | **Ref4** | **Ref5** | **Ref6** |
| 37 | 0.56 | 0.68 | 0.58 | 0.94 | 1.00 | 0.93 | 2 | 3 | 1 | 4 | 4 | 3 |
| 38 | 0.77 | 1.08 | 1.32 | 1.55 | 1.90 | 1.30 | 9 | 12 | 16 | 20 | 24 | 10 |
| 39 | 1.07 | 1.11 | 1.03 | 0.86 | 1.19 | 1.02 | 17 | 13 | 7 | 2 | 6 | 5 |
| 40 | 0.75 | 0.78 | 0.70 | 1.06 | 0.86 | 0.93 | 6 | 5 | 2 | 6 | 3 | 4 |
| 41 | 1.22 | 1.64 | 1.90 | 2.15 | 1.94 | 1.77 | 26 | 32 | 27 | 35 | 25 | 31 |
| 42 | 1.24 | 1.15 | 1.53 | 1.67 | 2.06 | 1.97 | 27 | 18 | 19 | 24 | 29 | 34 |
| 43 | 1.60 | 0.95 | 1.32 | 1.66 | 1.83 | 1.61 | 33 | 8 | 15 | 23 | 23 | 23 |
| 44 | 1.16 | 1.16 | 1.11 | 1.15 | 1.50 | 1.79 | 21 | 20 | 8 | 7 | 15 | 32 |
| 45 | 1.02 | 1.89 | 1.68 | 1.94 | 1.50 | 1.40 | 16 | 34 | 23 | 29 | 16 | 16 |
| 46 | 1.02 | 1.19 | 2.06 | 1.22 | 1.18 | 1.76 | 15 | 22 | 29 | 8 | 5 | 30 |
| 47 | 0.69 | 0.98 | 0.89 | 0.87 | 0.75 | 0.89 | 4 | 10 | 5 | 3 | 2 | 2 |
| 48 | 0.75 | 1.86 | 1.68 | 2.04 | 2.65 | 1.55 | 7 | 33 | 22 | 34 | 35 | 21 |
| 49 | 0.89 | 1.18 | 1.03 | 1.68 | 1.35 | 1.33 | 14 | 21 | 6 | 25 | 11 | 12 |
| 50 | 0.61 | 1.15 | 1.20 | 1.41 | 1.74 | 1.45 | 3 | 19 | 12 | 14 | 19 | 18 |
| 51 | 0.76 | 0.43 | 1.37 | 1.48 | 1.60 | 1.74 | 8 | 1 | 17 | 18 | 18 | 29 |
| 52 | 0.87 | 1.34 | 2.08 | 1.84 | 1.55 | 1.69 | 12 | 26 | 31 | 27 | 17 | 28 |
| 53 | 1.70 | 1.14 | 1.16 | 1.44 | 1.35 | 1.32 | 35 | 17 | 10 | 16 | 12 | 11 |
| 54 | 1.16 | 0.64 | 1.30 | 0.70 | 0.60 | 0.74 | 22 | 2 | 14 | 1 | 1 | 1 |
| 55 | 1.45 | 1.39 | 1.69 | 1.30 | 1.32 | 1.36 | 31 | 27 | 24 | 10 | 9 | 14 |
| 56 | 1.50 | 1.32 | 1.77 | 1.93 | 2.05 | 1.62 | 32 | 25 | 25 | 28 | 28 | 24 |
| 57 | 1.17 | 1.14 | 1.17 | 1.35 | 2.08 | 1.90 | 23 | 16 | 11 | 13 | 30 | 33 |
| 58 | 0.52 | 0.95 | 1.21 | 1.47 | 2.00 | 1.34 | 1 | 9 | 13 | 17 | 26 | 13 |
| 59 | 1.08 | 1.47 | 2.09 | 2.03 | 1.81 | 1.16 | 18 | 30 | 33 | 33 | 22 | 8 |
| 60 | 1.64 | 1.92 | 2.26 | 1.99 | 2.24 | 1.67 | 34 | 35 | 35 | 30 | 33 | 27 |
| 61 | 1.29 | 1.26 | 1.43 | 1.25 | 1.37 | 1.48 | 29 | 24 | 18 | 9 | 13 | 19 |
| 62 | 0.83 | 0.77 | 0.82 | 1.33 | 1.26 | 1.07 | 11 | 4 | 4 | 12 | 7 | 6 |
| 63 | 1.35 | 1.52 | 1.98 | 1.69 | 2.09 | 2.15 | 30 | 31 | 28 | 26 | 31 | 35 |
| 64 | 0.80 | 0.87 | 0.78 | 1.32 | 1.33 | 1.38 | 10 | 6 | 3 | 11 | 10 | 15 |
| 65 | 1.18 | 0.92 | 1.78 | 1.57 | 1.39 | 1.28 | 24 | 7 | 26 | 21 | 14 | 9 |
| 66 | 1.25 | 1.44 | 1.55 | 1.60 | 1.75 | 1.65 | 28 | 29 | 20 | 22 | 20 | 25 |
| 67 | 1.14 | 1.13 | 2.13 | 1.44 | 2.36 | 1.58 | 20 | 15 | 34 | 15 | 34 | 22 |
| 68 | 0.71 | 0.99 | 1.15 | 1.04 | 1.29 | 1.07 | 5 | 11 | 9 | 5 | 8 | 7 |
| 70 | 1.10 | 1.41 | 2.07 | 2.02 | 2.00 | 1.67 | 19 | 28 | 30 | 31 | 27 | 26 |
| 71 | 1.22 | 1.13 | 2.09 | 1.54 | 2.21 | 1.40 | 25 | 14 | 32 | 19 | 32 | 17 |
| 72 | 0.89 | 1.24 | 1.56 | 2.03 | 1.75 | 1.54 | 13 | 23 | 21 | 32 | 21 | 20 |
| **Value** | **Absolute response criterion** | | | | | | **Hierarchy response criterion** | | | | | |
| **ID** | **Ref1** | **Ref2** | **Ref3** | **Ref4** | **Ref5** | **Ref6** | **Ref1** | **Ref2** | **Ref3** | **Ref4** | **Ref5** | **Ref6** |
| 37 | 1,14 | 1,15 | 1,02 | 0,79 | 0,49 | 0,36 | 32 | 31 | 33 | 29 | 21 | 16 |
| 38 | 0,68 | 0,48 | 0,11 | -0,02 | -0,04 | 0,05 | 12 | 7 | 1 | 3 | 1 | 1 |
| 39 | 1,00 | 0,62 | 0,57 | 0,74 | 0,55 | 0,64 | 25 | 17 | 17 | 26 | 23 | 31 |
| 40 | 0,60 | 0,88 | 0,76 | 0,80 | 1,02 | 0,37 | 8 | 27 | 26 | 30 | 35 | 17 |
| 41 | 0,68 | 0,52 | 0,66 | 0,19 | 0,32 | 0,56 | 11 | 10 | 19 | 6 | 12 | 29 |
| 42 | 0,79 | 0,49 | 0,24 | -0,05 | 0,11 | 0,10 | 17 | 8 | 3 | 2 | 3 | 3 |
| 43 | 0,91 | 0,95 | 0,73 | 0,58 | 0,45 | 0,40 | 19 | 29 | 21 | 21 | 16 | 22 |
| 44 | 0,66 | 0,66 | 0,73 | 0,35 | 0,51 | 0,14 | 10 | 18 | 22 | 14 | 22 | 5 |
| 45 | 0,34 | 0,26 | 0,47 | 0,25 | 0,32 | 0,41 | 3 | 2 | 14 | 8 | 11 | 23 |
| 46 | 0,87 | 1,21 | 0,76 | 0,74 | 0,72 | 0,18 | 18 | 33 | 25 | 25 | 28 | 8 |
| 47 | 1,07 | 0,96 | 1,18 | 0,79 | 0,83 | 0,92 | 30 | 30 | 35 | 28 | 33 | 35 |
| 48 | 0,96 | 0,72 | 0,60 | 0,40 | 0,32 | 0,46 | 21 | 21 | 18 | 15 | 9 | 26 |
| 49 | 1,22 | 1,16 | 0,94 | 1,01 | 0,20 | 0,16 | 34 | 32 | 30 | 33 | 5 | 6 |
| 50 | 1,10 | 0,69 | 0,70 | 0,62 | 0,49 | 0,36 | 31 | 19 | 20 | 22 | 20 | 15 |
| 51 | 0,98 | 0,52 | 0,31 | 0,35 | 0,37 | 0,13 | 23 | 9 | 6 | 13 | 14 | 4 |
| 52 | 0,33 | 0,30 | 0,25 | -0,09 | 0,31 | 0,20 | 2 | 4 | 4 | 1 | 8 | 9 |
| 53 | 0,74 | 0,69 | 0,77 | 0,85 | 0,48 | 0,37 | 15 | 20 | 28 | 32 | 19 | 18 |
| 54 | 1,01 | 0,75 | 0,94 | 0,75 | 0,78 | 0,36 | 26 | 25 | 31 | 27 | 32 | 14 |
| 55 | 0,98 | 0,62 | 0,76 | 0,50 | 0,62 | 0,66 | 22 | 16 | 27 | 19 | 25 | 32 |
| 56 | 0,65 | 0,24 | 0,35 | 0,13 | 0,19 | 0,07 | 9 | 1 | 9 | 5 | 4 | 2 |
| 57 | 0,51 | 0,74 | 0,56 | 0,29 | 0,25 | 0,47 | 7 | 24 | 16 | 11 | 6 | 27 |
| 58 | 0,32 | 0,60 | 0,33 | 0,03 | 0,44 | 0,30 | 1 | 14 | 7 | 4 | 15 | 12 |
| 59 | 0,50 | 0,44 | 0,40 | 0,45 | 0,32 | 0,41 | 6 | 6 | 13 | 18 | 10 | 24 |
| 60 | 0,43 | 0,56 | 0,16 | 0,40 | 0,09 | 0,35 | 4 | 13 | 2 | 17 | 2 | 13 |
| 61 | 1,07 | 1,23 | 0,90 | 1,28 | 0,67 | 0,46 | 29 | 35 | 29 | 35 | 27 | 25 |
| 62 | 1,25 | 0,89 | 0,36 | 0,63 | 0,84 | 0,67 | 35 | 28 | 10 | 23 | 34 | 33 |
| 63 | 0,75 | 0,33 | 0,37 | 0,40 | 0,28 | 0,40 | 16 | 5 | 11 | 16 | 7 | 19 |
| 64 | 1,01 | 0,84 | 0,75 | 0,34 | 0,46 | 0,40 | 27 | 26 | 24 | 12 | 17 | 20 |
| 65 | 0,93 | 0,74 | 1,02 | 1,02 | 0,78 | 0,71 | 20 | 22 | 34 | 34 | 30 | 34 |
| 66 | 1,03 | 0,74 | 0,34 | 0,52 | 0,34 | 0,26 | 28 | 23 | 8 | 20 | 13 | 11 |
| 67 | 0,48 | 0,26 | 0,47 | 0,25 | 0,59 | 0,24 | 5 | 3 | 15 | 7 | 24 | 10 |
| 68 | 1,21 | 1,22 | 1,01 | 0,81 | 0,65 | 0,47 | 33 | 34 | 32 | 31 | 26 | 28 |
| 70 | 0,73 | 0,56 | 0,38 | 0,27 | 0,48 | 0,17 | 14 | 12 | 12 | 10 | 18 | 7 |
| 71 | 0,71 | 0,60 | 0,74 | 0,64 | 0,78 | 0,57 | 13 | 15 | 23 | 24 | 31 | 30 |
| 72 | 0,99 | 0,54 | 0,30 | 0,26 | 0,74 | 0,40 | 24 | 11 | 5 | 9 | 29 | 21 |
| **Value** | **Absolute premature response level values** | | | | | | **Hierarchy premature response level** | | | | | |
| **ID** | **Ref1** | **Ref2** | **ID** | **Ref1** | **Ref2** | **ID** | **Ref1** | **Ref2** | **ID** | **Ref1** | **Ref2** | **ID** |
| 37 | 23,20 | 37,12 | 21,83 | 25,85 | 37,40 | 46,64 | 20 | 30 | 24 | 25 | 33 | 34 |
| 38 | 23,74 | 38,10 | 41,56 | 24,78 | 31,15 | 44,89 | 22 | 31 | 35 | 23 | 26 | 33 |
| 39 | 23,54 | 44,26 | 37,40 | 30,70 | 47,12 | 42,00 | 21 | 33 | 34 | 30 | 35 | 31 |
| 40 | 38,13 | 32,14 | 34,05 | 28,24 | 30,59 | 44,59 | 31 | 26 | 32 | 28 | 25 | 32 |
| 41 | 9,43 | 16,64 | 12,41 | 26,31 | 16,08 | 21,40 | 4 | 7 | 8 | 26 | 8 | 9 |
| 42 | 12,89 | 27,75 | 26,62 | 17,79 | 14,19 | 19,64 | 10 | 20 | 28 | 11 | 5 | 6 |
| 43 | 9,63 | 15,92 | 14,89 | 19,54 | 16,37 | 27,37 | 5 | 5 | 13 | 15 | 10 | 17 |
| 44 | 23,09 | 19,84 | 21,48 | 36,85 | 18,36 | 14,80 | 18 | 11 | 23 | 34 | 12 | 2 |
| 45 | 52,53 | 27,04 | 13,52 | 26,50 | 33,71 | 30,42 | 35 | 18 | 10 | 27 | 29 | 21 |
| 46 | 25,45 | 8,72 | 7,39 | 21,64 | 30,04 | 22,38 | 24 | 2 | 2 | 19 | 24 | 12 |
| 47 | 26,31 | 39,10 | 14,17 | 21,68 | 27,75 | 20,09 | 26 | 32 | 12 | 20 | 22 | 7 |
| 48 | 20,84 | 15,18 | 12,37 | 11,00 | 22,17 | 28,53 | 17 | 4 | 7 | 3 | 15 | 20 |
| 49 | 15,23 | 9,06 | 21,00 | 10,05 | 36,78 | 34,71 | 11 | 3 | 20 | 2 | 31 | 24 |
| 50 | 18,93 | 29,63 | 31,55 | 36,02 | 28,27 | 34,34 | 14 | 23 | 31 | 33 | 23 | 23 |
| 51 | 19,91 | 57,64 | 24,03 | 19,41 | 31,87 | 27,08 | 16 | 35 | 25 | 14 | 28 | 15 |
| 52 | 40,20 | 29,47 | 16,53 | 24,77 | 37,23 | 28,08 | 33 | 22 | 16 | 22 | 32 | 18 |
| 53 | 15,55 | 19,67 | 12,17 | 12,10 | 27,19 | 24,32 | 12 | 10 | 6 | 4 | 21 | 14 |
| 54 | 9,73 | 33,89 | 24,33 | 33,43 | 38,08 | 51,08 | 6 | 28 | 26 | 31 | 34 | 35 |
| 55 | 11,03 | 25,19 | 13,72 | 14,63 | 16,19 | 23,25 | 7 | 16 | 11 | 5 | 9 | 13 |
| 56 | 18,12 | 29,44 | 16,36 | 24,81 | 21,58 | 39,12 | 13 | 21 | 15 | 24 | 14 | 28 |
| 57 | 25,19 | 35,97 | 17,24 | 30,32 | 16,94 | 22,15 | 23 | 29 | 17 | 29 | 11 | 11 |
| 58 | 45,02 | 47,26 | 34,85 | 44,17 | 34,28 | 34,85 | 34 | 34 | 33 | 35 | 30 | 26 |
| 59 | 38,79 | 22,40 | 8,10 | 20,66 | 24,10 | 36,11 | 32 | 14 | 3 | 17 | 17 | 27 |
| 60 | 19,17 | 17,68 | 19,66 | 16,05 | 20,81 | 19,06 | 15 | 9 | 19 | 7 | 13 | 5 |
| 61 | 11,44 | 6,85 | 21,04 | 20,51 | 15,40 | 31,62 | 8 | 1 | 21 | 16 | 7 | 22 |
| 62 | 11,88 | 26,56 | 29,05 | 18,11 | 14,92 | 19,04 | 9 | 17 | 30 | 12 | 6 | 4 |
| 63 | 29,71 | 32,01 | 16,05 | 22,08 | 25,96 | 18,98 | 28 | 25 | 14 | 21 | 20 | 3 |
| 64 | 26,54 | 20,48 | 28,02 | 35,08 | 31,20 | 28,11 | 27 | 12 | 29 | 32 | 27 | 19 |
| 65 | 8,11 | 21,20 | 5,77 | 3,18 | 8,70 | 13,86 | 2 | 13 | 1 | 1 | 1 | 1 |
| 66 | 9,16 | 16,47 | 13,04 | 15,25 | 25,16 | 34,77 | 3 | 6 | 9 | 6 | 19 | 25 |
| 67 | 26,11 | 31,76 | 11,81 | 17,23 | 11,85 | 21,95 | 25 | 24 | 5 | 9 | 3 | 10 |
| 68 | 7,72 | 17,28 | 19,21 | 16,20 | 24,96 | 41,22 | 1 | 8 | 18 | 8 | 18 | 30 |
| 70 | 34,74 | 23,15 | 21,35 | 21,59 | 22,88 | 40,82 | 30 | 15 | 22 | 18 | 16 | 29 |
| 71 | 23,11 | 33,06 | 9,69 | 18,89 | 12,00 | 27,21 | 19 | 27 | 4 | 13 | 4 | 16 |
| 72 | 31,49 | 27,08 | 25,47 | 17,30 | 11,84 | 20,29 | 29 | 19 | 27 | 10 | 2 | 8 |

**Table 10**: *Outcome of Pearson’s correlations analysis of the absolute and hierarchy reference (ref) values shown in Table 9. P-values were considered significant for P<0.05, and trend values for P<0.1. N=35*

| **Discriminability (d’)** | | | | | | |
| --- | --- | --- | --- | --- | --- | --- |
| **Absolute d’** | **Ref1** | **Ref2** | **Ref3** | **Ref4** | **Ref5** | **Ref6** |
| **Ref1** |  |  |  |  |  |  |
| **Ref2** | 0.38, P<0.05 |  |  |  |  |  |
| **Ref3** | 0.46, P<0.01 | 0.63, P<0.001 |  |  |  |  |
| **Ref4** | 0.27, P=0.123 | 0.68, P<0.001 | 0.65, P<0.001 |  |  |  |
| **Ref5** | 0.24, P=0.158 | 0.55, P<0.001 | 0.59, P<0.001 | 0.73, P<0.001 |  |  |
| **Ref6** | 0.39, P<0.05 | 0.44, P<0.01 | 0.54, P<0.001 | 0.57, P<0.001 | 0.68, P<0.001 |  |
| **Hierarchy d’** | **Ref1** | **Ref2** | **Ref3** | **Ref4** | **Ref5** | **Ref6** |
| **Ref1** |  |  |  |  |  |  |
| **Ref2** | 0.41, P<0.05 |  |  |  |  |  |
| **Ref3** | 0.46, P<0.01 | 0.64, P<0.001 |  |  |  |  |
| **Ref4** | 0.28, P=0.102 | 0.67, P<0.001 | 0.64, P<0.001 |  |  |  |
| **Ref5** | 0.33, P=0.050 | 0.49, P<0.01 | 0.63, P<0.001 | 0.71, P<0.001 |  |  |
| **Ref6** | 0.42, P<0.05 | 0.52, P<0.01 | 0.50, P<0.01 | 0.48, P<0.01 | 0.64, P<0.001 |  |
| **Response criterion (C)** | | | | | | |
| **Absolute C** | **Ref1** | **Ref2** | **Ref3** | **Ref4** | **Ref5** | **Ref6** |
| **Ref1** |  |  |  |  |  |  |
| **Ref2** | 0.67, P<0.001 |  |  |  |  |  |
| **Ref3** | 0.52, P<0.001 | 0.70, P<0.001 |  |  |  |  |
| **Ref4** | 0.61, P<0.001 | 0.75, P<0.001 | 0.78, P<0.001 |  |  |  |
| **Ref5** | 0.35, P<0.05 | 0.43, P<0.01 | 0.58, P<0.001 | 0.58, P<0.001 |  |  |
| **Ref6** | 0.30, P=0.083 | 0.25, P=0.147 | 0.50, P<0.01 | 0.46, P<0.01 | 0.55, P<0.001 |  |
| **Hierarchy C** | **Ref1** | **Ref2** | **Ref3** | **Ref4** | **Ref5** | **Ref6** |
| **Ref1** |  |  |  |  |  |  |
| **Ref2** | 0.67, P<0.001 |  |  |  |  |  |
| **Ref3** | 0.47, P<0.01 | 0.71, P<0.001 |  |  |  |  |
| **Ref4** | 0.60, P<0.001 | 0.77, P<0.001 | 0.79, P<0.001 |  |  |  |
| **Ref5** | 0.40, P<0.05 | 0.50, P<0.01 | 0.59, P<0.001 | 0.58, P<0.001 |  |  |
| **Ref6** | 0.27, P=0.121 | 0.33, P=0.057 | 0.46, P<0.01 | 0.43, P<0.01 | 0.48, P<0.01 |  |
| **Premature response level (%PR)** | | | | | | |
| **Absolute %PR** | **Ref1** | **Ref2** | **Ref3** | **Ref4** | **Ref5** | **Ref6** |
| **Ref1** |  |  |  |  |  |  |
| **Ref2** | 0.35, P<0.05 |  |  |  |  |  |
| **Ref3** | 0.14, P=0.434 | 0.45, P<0.01 |  |  |  |  |
| **Ref4** | 0.43, P<0.01 | 0.42, P<0.01 | 0.54, P<0.001 |  |  |  |
| **Ref5** | 0.35, P<0.05 | 0.34, P<0.05 | 0.42, P<0.01 | 0.44, P<0.01 |  |  |
| **Ref6** | 0.12, P=0.496 | 0.21, P=0.220 | 0.44, P<0.01 | 0.32, P=0.061 | 0.63, P<0.001 |  |
| **Hierarchy %PR** | **Ref1** | **Ref2** | **Ref3** | **Ref4** | **Ref5** | **Ref6** |
| **Ref1** |  |  |  |  |  |  |
| **Ref2** | 0.43, P<0.01 |  |  |  |  |  |
| **Ref3** | 0.14, P=0.407 | 0.44, P<0.01 |  |  |  |  |
| **Ref4** | 0.44, P<0.01 | 0.48, P<0.01 | 0.49, P<0.01 |  |  |  |
| **Ref5** | 0.34, P<0.05 | 0.33, P<0.05 | 0.37, P<0.05 | 0.47, P<0.01 |  |  |
| **Ref6** | 0.10, P=0.551 | 0.19, P=0.278 | 0.34, P<0.05 | 0.31, P=0.075 | 0.62, P<0.001 |  |

**Supplementary references**

Arnsten AFT and Dudley AG (2005) Methylphenidate improves prefrontal cortical cognitive function through α2 adrenoceptor and dopamine D1 receptor actions: Relevance to therapeutic effects in Attention Deficit Hyperactivity Disorder. Behavioral and Brain Functions, 1, pp. 1–9. doi: 10.1186/1744-9081-1-2.

Barlow RL, Dalleyn JW and Pekcec A (2018) Differences in trait impulsivity do not bias the response to pharmacological drug challenge in the rat five-choice serial reaction time task. Psychopharmacology. Psychopharmacology, 235(4), pp. 1199–1209. doi: 10.1007/s00213-018-4836-5.

Cumming P (2002) Specific Binding of [11C] Raclopride and N-[3H]Propyl-Norapomorphine to Dopamine Receptors in Living Mouse Striatum: Occupancy by Endogenous Dopamine and Guanosine Triphosphate–Free G Protein. J Cereb Blood Flow Metab, 22(5), pp. 596-604. doi: 10.1097/00004647-200205000-00011

Grimm CM (2018) Schizophrenia-related cognitive dysfunction in the Cyclin-D2 knockout mouse model of ventral hippocampal hyperactivity. Translational Psychiatry, 8(1), p. 212. doi: 10.1038/s41398-018-0268-6.

Haile CN (2012) The α1 antagonist doxazosin alters the behavioral effects of cocaine in rats. Brain Sciences, 2(4), pp. 619–633. doi: 10.3390/brainsci2040619.

Mahoney MK (2016) Pharmacological investigations of a yohimbine-impulsivity interaction in rats. Behavioural Pharmacology, 27(7), pp. 585–595. doi: 10.1097/FBP.0000000000000251

Mantsch JR (2010) Involvement of Noradrenergic Neurotransmission in the Stress- but not Cocaine-Induced Reinstatement of Extinguished Cocaine-Induced Conditioned Place Preference in Mice: Role for β-2 Adrenergic Receptors. Neuropsychopharmacology, 35(11), pp. 2165–2178. doi: 10.1038/npp.2010.86.

Xueliang Fan and Hess EJ (2008) D2-like dopamine receptors mediate the response to amphetamine in a mouse model of ADHD. Neurobiol Dis, 26(1), pp. 201–211.
